# MSK1 promotes colorectal cancer metastasis by increasing Snail protein stability through USP5-mediated Snail deubiquitination

**DOI:** 10.1038/s12276-025-01433-0

**Published:** 2025-04-01

**Authors:** Keun-Seok Hong, Ki-Jun Ryu, Hyemin Kim, Minju Kim, Seung-Ho Park, Taeyoung Kim, Jung Wook Yang, Cheol Hwangbo, Kwang Dong Kim, Young-Jun Park, Jiyun Yoo

**Affiliations:** 1 Department of Bio and Medical Bigdata (Brain Korea 21 Four), Gyeongsang National University, Jinju, Republic of Korea; 2 Anti-aging Bio Cell Factory Regional Leading Research Center, Gyeongsang National University, Jinju, Republic of Korea; 3 Department of Biochemistry and Institute of Health Sciences, Gyeongsang National University School of Medicine, Jinju, Republic of Korea; 4 Division of Applied Life Science (Brain Korea 21 Four), Research Institute of Life Sciences, Gyeongsang National University, Jinju, Republic of Korea; 5 Environmental Disease Research Center, Korea Research Institute of Bioscience and Biotechnology, Daejeon, Republic of Korea; 6 Department of Pathology, Gyeongsang National University Hospital, Gyeongsang National University College of Medicine, Jinju, Republic of Korea; 7 Division of Life Science, College of Natural Sciences, Gyeongsang National University, Jinju, Republic of Korea

**Keywords:** Metastasis, Phosphorylation, Ubiquitylation

## Abstract

Mitogen- and stress-activated protein kinase 1 (MSK1), a Ser/Thr kinase, phosphorylates nuclear proteins to increase their stability and DNA-binding affinity. Despite the role of MSK1 in promoting cancer progression in colorectal cancer (CRC), the precise molecular mechanisms remain unelucidated. Here we show that MSK1 expression induces the epithelial–mesenchymal transition (EMT) process and increases CRC cell metastasis. Furthermore, we discovered that MSK1 interacts with Snail, a key EMT regulator, and increases its stability by inhibiting ubiquitin-mediated proteasomal degradation. Importantly, MSK1 increased Snail protein stability by promoting deubiquitination rather than inhibiting its ubiquitination. Finally, we identified USP5 as an essential deubiquitinase that binds to Snail protein phosphorylated by MSK1. Based on the experimental data, in CRC, MSK1–Snail–USP5 axis can promote EMT and metastasis of CRC. Together, our findings provide potential biomarkers and novel therapeutic targets for further research in CRC.

## MSK1 enhances EMT and metastasis in colorectal cancer

Colorectal cancer (CRC) is a major health concern worldwide, often leading to death due to its spread to other body parts. To find better treatments, researchers are trying to understand how CRC spreads and have focused on a protein called MSK1, which might play a role in this process. The study used human cancer cell lines and mice to explore how MSK1 affects another protein called Snail, which is involved in cancer spread. They found that MSK1 helps to stabilize Snail by preventing its breakdown, which, in turn, promotes the spread of cancer cells. This was done by examining the interaction between MSK1 and Snail in cells and observing changes in cancer cell behavior. The results showed that high levels of MSK1 are linked to increased cancer spread and lower survival rates in patients with CRC.

This summary was initially drafted using artificial intelligence, then revised and fact-checked by the author.

## Introduction

Colorectal cancer (CRC) is the third most commonly diagnosed cancer and the second leading cause of cancer-related mortality worldwide, accounting for approximately one million deaths annually^1^. Although most patients with early-stage CRC can be cured surgically, the 5-year survival rate is significantly reduced when distant tumor metastasis occurs^2^. Over the past 20 years, advances in surgical techniques and novel targeted therapy combinations have effectively extended the median survival of patients with metastatic CRC. However, CRC mortality remains strongly associated with metastasis^3^. Thus, a better understanding of the malignant progression of CRC will aid in developing potential targets and therapeutic opportunities.

Primary CRC originates from epithelial cells that line the gastrointestinal tract. Distant metastasis occurs in approximately 25% of patients and is a major cause of poor prognosis and mortality in patients with CRC. During metastasis, epithelial cells lose apical–basal polarity and cell–cell contact, resulting in a mesenchymal phenotype^4^. This loss of epithelial features is often accompanied by increased cell motility and the expression of mesenchymal genes, a process known as the epithelial–mesenchymal transition (EMT) and considered a key step in the progression of tumors toward metastasis^5–7^. These changes enable tumor cells to migrate through the extracellular matrix and colonize lymph/blood vessels, thereby initiating the metastatic cascade^8^. Thus, EMT regulators, which probably play crucial roles in cancer progression, have been extensively studied.

Aberrant regulation of EMT-related transcription factors, such as Snail, Slug, Twist and Zeb, has been identified in CRC and is associated with invasiveness, metastasis and poor prognosis^9–14^. One such regulator is the zinc-finger protein Snail, which induces EMT by directly repressing E-cadherin transcription during tumor development or progression^15,16^. Snail is a highly unstable protein, and its subcellular levels or protein stability are primarily regulated by various kinases. For example, GSK3β-mediated Snail phosphorylation negatively regulates Snail function by inducing nuclear export and ubiquitination-dependent cytosolic degradation^17–19^. By contrast, other kinases positively regulate Snail function by inducing nuclear import and retention and increasing its stability^20–25^.

Mitogen- and stress-activated protein kinase 1 (MSK1) is a member of the MAPK-activated protein kinase subfamily that is activated by extracellular signal-regulated kinases 1 and 2 (Erk1/2) and p38 MAPKs in response to mitogenic signals or cellular stresses^26–29^. Many transcription factors, including the cAMP-response element-binding (CREB) protein, activating transcription factor 1 (ATF1) and the p65 subunit of nuclear factor kappa B (NF-κB), have been identified as substrates of MSK1 (refs. ^26,30,31^). MSK1 phosphorylation of these transcription factors may alter their ability to bind to their target DNA and/or recruit required coactivators. MSK1 phosphorylates chromatin proteins histone H3, a nucleosome component, and high-mobility group nucleosome binding domain 1 (HMGN1; HMG-14), a chromatin-associated protein^32,33^. MSK1 performs various physiological functions in cells; however, conflicting results regarding its role in cancer development and metastasis have been published^34–40^. According to some studies, MSK1 is essential for steroid-hormone-induced breast cancer cell proliferation^38^, whereas others indicate that elevated MSK1 expression is associated with a higher survival rate in patients with breast cancer^39^. MSK1 expression also inhibits cancer metastasis in luminal breast cancer by activating the luminal gene via histone H3 phosphorylation^40^.

MSK1 is essential for tumor aggressiveness and metastatic potential in CRC^41^; however, the underlying molecular mechanism remains unknown. Therefore, we investigated the role of MSK1 in mediating Snail protein stability and its associated molecular mechanisms in regulating EMT and colorectal tumor metastasis.

## Materials and methods

### Cell culture

All cell lines used in this study were obtained from the Korean Cell Line Bank, where they were characterized by DNA fingerprinting and isozyme detection and cultured according to American Type Culture Collection instructions. All cell lines were used within 3–20 passages of thawing the original stocks and were tested every 3 months for mycoplasma contamination. The cell lines were maintained for no more than three passages between experiments. Human HEK293T and human CRC cell lines (SW480, Caco2, SW620, HCT116) were cultured in Dulbecco’s modified Eagle medium (DMEM; Invitrogen) supplemented with 10% fetal bovine serum (FBS) and 1% penicillin and streptomycin.

### Plasmid construction and transfection

The wild-type (WT) MSK1, Snail and USP5 plasmids were purchased from Sino Biological. Site-directed mutagenesis was performed with a QuikChange mutagenesis kit (Stratagene), according to the manufacturer’s instructions. For transient transfection, HEK293T cells were seeded in a six-well or 100-mm-diameter dish for 24 h and transfected with the indicated plasmid by using X-tremeGENE HP DNA transfection reagent (Roche) following the manufacturer’s instructions. After 48 h, the cells were collected and used for western blot analysis. Two different small interfering RNA (siRNA) oligo duplexes for targeting human MSK1 (siMSK1-1; 5′-GAGCAUGAGGUGCAGAUUUAU-3′ and siMSK1-2; 5′-AUAAAUCUGCACCUCAUGCUC-3′) or USP5 (siUSP5-1; 5′-GGGAAACAGUAUGUGGAGAGA-3′ and siUSP5-2; 5′-GGCCACAGAGAAGGUGAAGUA-3′), respectively, were purchased from Bioneer. Transient transfection of siRNA oligo duplex was accomplished using Lipofectamine 3000 (Invitrogen) following the manufacturer’s instructions. For stable transfection, SW480 cells were transfected with plasmid expressing Flag-tag (control), Flag-WT-MSK1, Flag-K455M-MSK1 or Flag-D565A-MSK1 by using the X-tremeGENE HP DNA transfection reagent (Roche). After 48 h incubation, 200 μg/ml of Hygromycin B Gold (InvivoGen) was added to the cultures to select for hygromycin-resistant clones. After 3–4 weeks, independent colonies were picked using cloning cylinder (Sigma), subcultured and tested for MSK1 expression by western blot analysis. Two different short hairpin (sh)RNA expression vectors for targeting human Snail (shSnail-1; 5′-GCAAATACTGCAACAAGGAAT-3′ and shSnail-2; 5′-CCAAGGATCTCCAGCTCGAA-3′) were purchased from Sigma-Aldrich. shRNA expression vector was transfected into lentiviral packaging cell lines 293T cells. The culture supernatant containing virus particles was collected 48 h after transfection, clarified with a 0.45 μM membrane filter. For stable transduction of lentivirus containing shRNA expression vector, cells at 60–70% confluency were grown in six-well plates, and 1 ml of viral supernatant containing 4 μg of polybrene was added. After 48 h, 1 μg/ml puromycin (Gibco) was added to the cultures for selection. After 14 days, puromycin-resistant cell pools were established.

### Immunoprecipitation

Cells were lysed in lysis buffer (20 mM Tris pH 7.4, 2 mM EDTA, 150 mM sodium chloride, 1 mM sodium deoxycholate, 1% Triton X-100, 10% glycerol and two pills of protease inhibitor cocktail (Roche)), mixed by vortexing and incubated 30 min on ice. Lysates were precleared using protein A/G beads (Santa Cruz), incubated with the specific antibodies for overnight at 4 °C and then incubated with beads for 2 h at 4 °C with gentle mixing. Beads were then washed five times with lysis buffer and eluted with 30 μl of 2× SDS sample buffer. Western blot analysis was then performed. Primary antibodies used for immunoprecipitation (IP) were as follows: anti-Flag (Abm G191; 2 μg/ml), anti-HA (Abm G036; 4 μg/ml), anti-Myc (Proteintech no. 60003-2-1lg; 2 μg/ml) and anti-Snail (Cell Signaling no. 3879; 5 μg/ml).

### Western blot analysis

Protein samples were subjected to SDS–PAGE and transferred to polyvinylidene fluoride membranes. Membranes were incubated with indicated primary antibodies overnight at 4 °C. After washing with TBS-T (TBS containing 0.1% Tween-20), membranes were incubated with corresponding horseradish-peroxidase-conjugated secondary antibodies (1:5,000) for 1 h at room temperature. Blots were developed with enhanced chemiluminescence (Bio-Rad) reaction according to the manufacturer’s instructions. Primary antibodies used were: anti-MSK1 (Cell Signaling, no. 3489; 1:1,000), anti-phospho-MSK1(Thr581) (Cell Signaling, no. 9595; 1:1,000), anti-α-tubulin (Sigma-Aldrich, T6199, 1:10,000), anti-Flag (Abm, G191; 1:2,000), anti-Snail (Cell Signaling, no. 3879; 1:1,000), anti-Slug (Cell Signaling, no. 9585; 1:1,000), anti-Twist (Abcam, ab49254; 1:1,000), anti-fibronectin (BD Biosciences, no. 610078; 1:5,000), anti-N-cadherin (BD Biosciences, no. 610921; 1:5,000), anti-vimentin (Santa Cruz Biotechnology, sc-6260; 1:1,000), anti-E-cadherin (BD Biosciences, no. 610181; 1:10,000), anti-occludin (Thermo Fisher Scientific, no. 71-1500; 1:1,000), anti-claudin-1 (Cell Signaling, no. 13995; 1:1,000), anti-HA (Abm, G036; 1:2,000), anti-HA High Affinity (Roche, no. 11867423001; 1:1,000), anti-Myc (Proteintech, no. 60003-2lg; 1:2,000), anti-ubiquitin (Santa Cruz Biotechnology, sc-8017; 1:2,000), anti-phospho-histone H3 (Cell Signaling, no. 53348; 1:1,000), anti-histone H3 (Cell Signaling, no. 4499; 1:1,000), anti-phospho-serine (Abcam, ab9334; 1:1,000) and anti-USP5 (Proteintech, no. 10473-1-AP; 1:1,000).

### Total RNA extraction and qRT–PCR

Total RNA was extracted from the cultured cells using RNeasy Mini Kit (Qiagen) following the manufacturer’s instructions. Reverse transcription was performed using iScript cDNA synthesis kit (Bio-Rad) following the manufacturer’s instructions. qRT–PCR (quantitative reverse transcription-polymerase chain reaction) was performed using iQ SYBRq RT/PCR PreMix (Bio-Rad) following the manufacturer’s instructions. Amplification was performed using CFX96 Touch Real-Time Detection System. Primers used were as follows: Snail (F: 5′-ATCGGAAGCCTAACTACAGC-3′; R: 5′-CAGAGTCCCAGATGAGCATT-3′), GAPDH (F: 5′-GTGGTCTCCTCTGACTTCAAC-3′; R: 5′-TCTCTTCCTCTTGTGCTCTTG-3′).

### In vitro kinase assay

The in vitro kinase assay was conducted using two different methodologies, depending on the time frame of the experiments. Before 2022, the in vitro kinase assay was performed by incubating recombinant active MSK1 (Millipore) kinase protein with purified MBP-fused WT Snail protein, which was expressed in pDEST-MBP vector in *Escherichia coli*, in kinase buffer (25 mM Tris–HCl pH 7.5, 5 mM β-glycerophosphate, 2 mM dithiothreitol (DTT), 0.1 mM Na_3_VO_4_ and 10 mM MgCl_2_) in the presence or absence of [γ-^32^P]-ATP (BMS). The reaction was carried out for 30 min at 30 °C and was terminated with SDS sample buffer. Each sample was then boiled for 10 min at 100 °C, followed by SDS–PAGE and autoradiography. Due to changes in legislation restricting the use of radioactive isotopes in 2022, we adapted our methodology to use ATP-γ-S as a nonradioactive alternative. The in vitro kinase assay was performed by incubating recombinant active MSK1 (Millipore) kinase protein and recombinant Snail protein (abcam) in the presence of ATP-γ-S (abcam) and 200 μM cold ATP for 1 h at 30 °C in kinase buffer (50 mM Tris–HCl pH 7.5, 10 mM MgCl_2_ and 2 mM DTT). Thiophosphorylation of the substrate by the MSK1 was stopped by adding a final concentration of 0.1 mM EDTA. The thiophosphorylated substrate was alkylated with final concentration of 2.5 mM *p*-nitrobenzyl mesylate (abcam) for 1.5 h at room temperature, resulting in the generation of a thiophosphate ester at the thiophosphorylated residue of the substrate. The reaction was stopped by adding SDS sample buffer and then subjected to western blot analysis using an anti-thiophosphate ester antibody (abcam).

### Construction and purification of MBP-fusion protein

The WT MBP-fused Snail plasmid was purchased from Sino Biological. Site-directed mutagenesis was performed with a QuikChange mutagenesis kit (Stratagene), according to the manufacturer’s instructions. Each MBP-fused Snail construct was transformed into *E. coli* BL21(DE3) cells. The cells were grown until the OD_600_ (optical density of a sample measured at a wavelength of 600 nm in 1 cm light path) reached 0.6–0.8. Protein expression was induced with 0.5 mM isopropyl β-d-1-thiogalactopyranoside at 16 °C for 16 h. Bacterial cells were collected by centrifugation and resuspended in lysis buffer (50 mM Tris–HCl pH 7.5, 150 mM NaCl, 1 mM EDTA, 0.5 mM DTT, 0.1% Triton X-100 and 1 mM phenylmethylsulfonyl fluoride). Cells were lysed by sonication on ice, and the lysate was clarified by centrifugation. MBP-fused Snail protein present in the supernatant was purified using the MBP Excellose Spin Kit (Bioprogen) according to the manufacturer’s instructions.

### Cycloheximide pulse-chase assay

HEK293T, SW480, Caco2, SW620 and HCT116 cells were seeded on a 12-well plate at a density of 5 × 10^5^ cells per well. After culturing overnight, the cells were transfected with plasmids or siRNAs as desired. Two days after transfection, the cells were treated with 100 μg/ml of cycloheximide (CHX). Total protein lysates were collected at different time points and subjected to immunoblotting for HA or Snail.

### Ubiquitination assay

Ubiquitination assay was done following an IP protocol. HEK293T cells were transfected with HA–Ub, Myc–Snail and Flag–MSK1. Two days after transfection, cells were treated with 10 μM MG132 for 6 h to block proteasomal degradation of the Snail protein before lysing with Triton X-100 lysis buffer. Cell lysates were then collected and immunoprecipitated with anti-Myc antibody (Proteintech, no. 60003-2-1lg; 2 μg/ml) to specifically pull down Myc–Snail protein. Pulled-down samples were subject to immunoblotting with anti-HA (ubiquitin) to visualize polyubiquitinated Snail protein bands.

### Proliferation assay

Cells were seeded in six-well plates at 1 × 10^5^ cells per well. After incubation for 1–4 days, cells were trypsinized and resuspended in 1 ml of appropriate medium. The viable cells were stained with trypan blue and counted with a hemocytometer.

### Migration assay

Cells were seeded into Culture-Insert (Ibidi) at 5.0 × 10^5^ cells per insert. After the cells were confluent, the Culture-Insert was removed and washed with phosphate-buffered saline three times to rinse off the detached cells. Cells were then cultured with appropriate fresh media for further 24 h. The wound closure was observed and photographed at indicated times, using a phase-contrast microscope with a digital camera.

### Invasion assay

Invasion assays were assessed using QCM 24-Well Cell Invasion Assay (Fluorometric) kit (Millipore) following the manufacturer’s instructions. Cells were serum-starved for 24 h, and 2.5 × 10^5^ cells in 250 μl of serum-free medium were seeded into upper chambers. The lower chambers were filled with 500 μl of appropriate media containing 20% fetal bovine serum. Thirty hours after incubation, noninvaded cells or medium remaining on the upper chambers were removed by pipetting. The upper chambers were transferred into a clean well containing 225 μl of prewarmed cell detachment solution and incubated for 30 min at 37 °C. The upper chambers were removed from the well. Then, 75 μl of lysis buffer/dye solution (CyQuant GR dye 1:75 with 4× lysis buffer) was added into each well and incubated for 15 min at room temperature. Two hundred microliters of the mixture were transferred into a 96-well plate and assessed with a fluorescence plate reader using a 480/520 nm filter set.

### Mice and animal housing

Male BALB/c nude mice at 4–5 weeks of age were purchased from DooYeol Biotech and housed in a pathogen-free barrier room in Animal Care Facility at Korea Research Institute of Bioscience and Biotechnology (KRIBB). All experiments using animals were conducted under the Institutional Animal Care and Use Committee-approved protocols (approval number KRIBB-AEC-21135) at KRIBB in accordance with institutional guidelines.

### Xenograft studies

For metastasis analysis of SW480 human CRC models, 2 × 10^6^ of SW480(Con), WT MSK1 or mutant MSK1-expressing SW480(WT-MSK1), SW480(K455M-MSK1), SW480(D565A-MSK1) or Snail-depleting in WT MSK1-expressing SW480(MSK1/shSnail-1 and MSK1/shSnail-2) cells were injected intravasationally into BALB/c male nude mice (*n* = 5 for each group). Ten weeks after the injection, the mice were euthanized and the lungs were stained with hematoxylin and eosin (H&E).

### CRC tissue specimens

Deidentified and paraffin-embedded human CRC tissue specimens (78 cases) were collected at Gyeongsang National University Hospital, Jinju, Korea. These clinical CRC tissue specimens were examined and diagnosed by pathologists in 2012 at Gyeongsang National University Hospital. This study was approved (approval number 2018-04-010) by the institutional review board at Gyeongsang National University Hospital with a waiver of informed consent.

### Tissue microarray and immunohistochemistry

We used tissue microarray (TMA) for immunohistochemistry (IHC). Three-millimeter-diameter core tissues were obtained from individual formalin-fixed and paraffin-embedded tissue, and arranged in new recipient paraffin blocks. Two tissue cores from the most representative tumor areas were analyzed. IHC was performed on 4-μm-thick TMA paraffin sections using a BenchMark ULTRA (Ventana Medical Systems) and Optiview DAB IHC Detection Kit (Ventana Medical Systems). Polyclonal antibodies specific to Snail (1:750) and phospho-MSK1(Thr581) (Cell Signaling no. 9595; 1:50) were used for IHC. ULTRA Cell Conditioning 1 (Ventana Medical Systems) was used (56 min, 37 °C) for antigen retrieval for Snail and phospho-MSK1. The incubation time for primary antibodies was 32 min.

The expression of each protein was examined with blindness to each IHC result. Snail and phospho-MSK1 were expressed in nucleus. Staining intensity was scored as 0 (negative), 1 (mild), 2 (moderate) or 3 (marked). The proportional score of stained tumor cells was classified into 0 (<5%), 1 (5–25%), 2 (26–50%), 3 (51–75%) and 4 (76–100%). Expression score was calculated by multiplying the intensity score by the proportional score (0–12). An expression score higher than 4 was considered high expression; if lower than 4, it was considered low expression.

### Heat map

The dataset (GSE 40967) was stratified on the basis of the expression levels of MSK1, after which five sets of samples were randomly selected from both the high and low MSK1 expression groups. The low MSK1 expression group comprised samples CIT226, CIT129, CIT115, CIT084 and CIT004, while the high MSK1 expression group consisted of samples CIT184, CIT482, CIT276, CIT299 and CIT133. Subsequently, the expression levels of EMT markers (N-cadherin, vimentin, fibronectin, α-SMA, E-cadherin, claudin-1 and occludin) in each sample were normalized to values ranging from 0 to 100. A heat map was then generated using these normalized values.

### Statistical analysis

Quantitative data in this study are presented as mean ± s.d. and were analyzed by Student’s *t*-test, one-way analysis of variance (ANOVA) or two-way ANOVA. *P* < 0.05 was considered statistically significant. For the quantification of protein stability after treatment of CHX, Snail and α-tubulin proteins detected by immunoblotting were quantified using ImageJ software. For normalization, α-tubulin expression was used as a control. GraphPad Prism version 7 and SPSS version 26 software were used in this study. All experiments were repeated at least three independent times. Animal studies were performed with adequate *n* numbers to ensure statistical evaluation. No statistical method was used to predetermine sample size. Sample size was chosen on the basis of literature in the field.

## Results

### MSK1 promotes CRC cell metastasis by inducing the EMT process

**Fig. 1:**
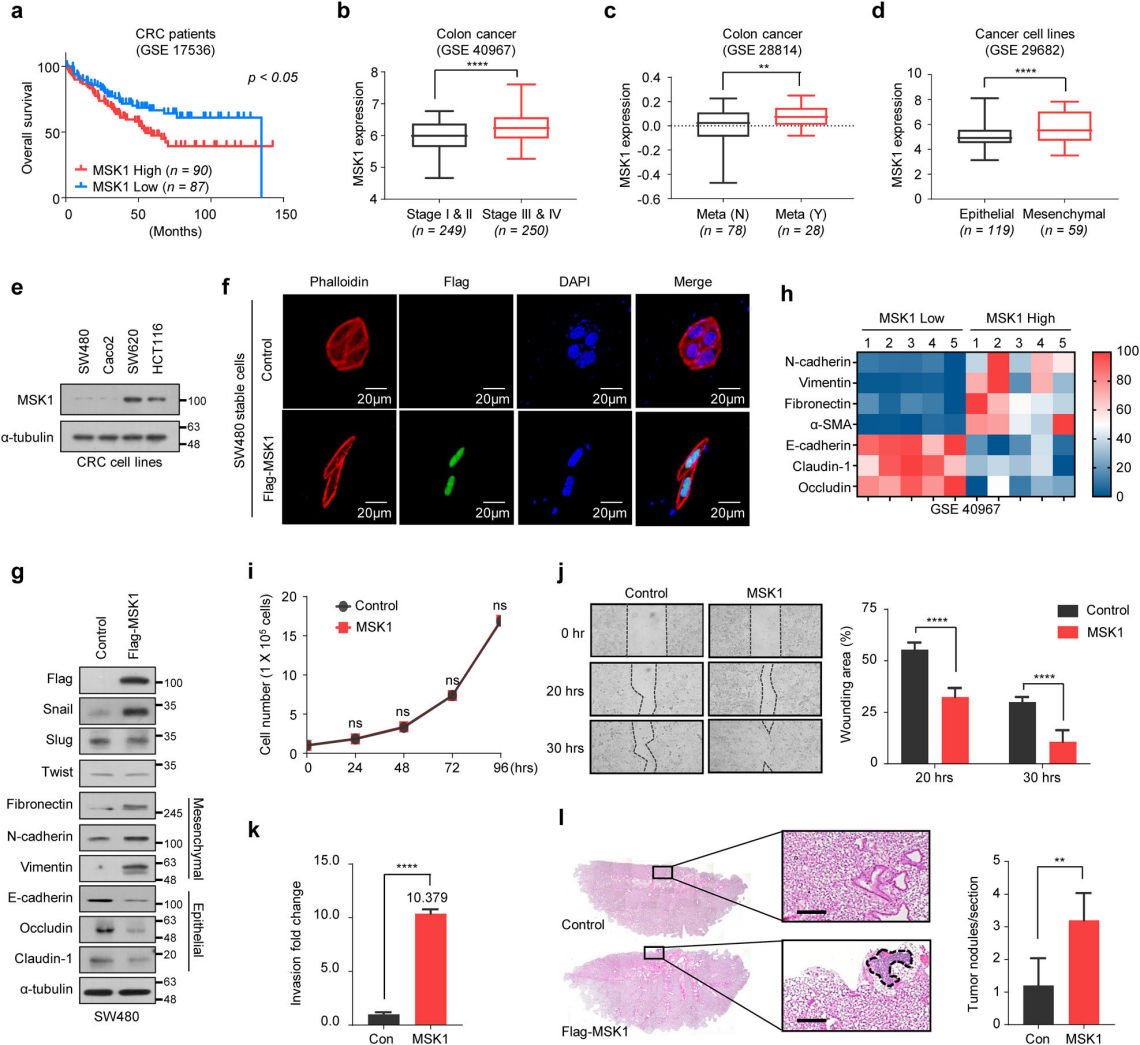
MSK1 promotes CRC cell metastasis by inducing the EMT process. **a** The association of MSK1 expression with overall survival in patients with CRC. The Kaplan–Meier plot shows the reduced survival probability of patients with high levels of MSK1 mRNA (*n* = 90) compared with low MSK1 mRNA levels (*n* = 87) (GSE 17536). Statistical analysis was performed using log-rank tests. **b** The association of MSK1 expression with tumor stages in patients with CRC. The box plot shows increased MSK1 mRNA levels in patients with late-stage CRC (stages III and IV, *n* = 250) compared with patiehts with early-stage CRC (stages I and II, *n* = 249) (GSE 40967). ****P < 0.0001 as determined by *t*-test. **c** The association of MSK1 expression with metastasis in patients with CRC. The box plot shows increased MSK1 mRNA levels in patients with metastasis (*n* = 28) compared with patients without metastasis (*n* = 78) (GSE 28814). **P < 0.01 as determined by *t*-test. **d** The association of cancer cell types with MSK1 expression. The box plot shows increased MSK1 mRNA levels in mesenchymal cancer cells (*n* = 59) compared with epithelial cancer cells (*n* = 119) (GSE 29682). ****P < 0.0001 as determined by *t*-test. **e** Expression levels of MSK1 protein in various CRC cell lines (SW480 and Caco2, epithelial; SW620 and HCT116, mesenchymal) analyzed by immunoblotting. **f** Morphological changes of MSK1-expressing SW480 cells. MSK1-overexpressing SW480 and control cells are visualized by confocal microscopy after staining with tetramethylrhodamine-isothiocyanate (TRITC)-conjugated phalloidin. Scale bars, 20 µm. **g** Expression levels of EMT marker proteins in MSK1-expressing SW480 and control cells analyzed by immunoblotting. **h** A heat map showing the relative expression levels of EMT marker genes in patients with CRC with high MSK1 expression and low MSK1 expression. Patients with high or low MSK1 expression were randomly extracted from the microarray dataset (GSE 40967) and analyzed. **i** The indicated cells were seeded in a six-well plate at a concentration of 1 × 10^5^ cells per well. After incubation for 1–4 days, the viable cells were counted with a hemocytometer after trypan blue staining**. j** Left: MSK1-overexpressing SW480 and control cells were analyzed in wound-healing assays by visualizing wound closure via phase-contrast microcopy. Wound areas were measured using WimScratch software (Wimasis). Right: the data shown represent the percentage of the wound area and are expressed as the mean ± s.d. of three individual experiments. ****P < 0.0001 as determined by *t*-test. **k** MSK1-overexpressing SW480 and con*t*rol cells were seeded onto Matrigel matrix-coated top chambers, and the fold changes of invading cells were measured after 30 h. The data shown are expressed as the mean ± s.d. of three individual experiments, each performed in triplicate. ****P < 0.0001 as determined by *t*-test. **l** About 2 × 10^6^ of the SW480 cells stably overexpressing MSK1 or control cells were injected into the nude mice by tail-vein injection. Left: representative pictures of H&E staining of lung sections. Scale bars, 100 μm. Right: the number of metastatic lung nodules in individual mice was quantified at 10 weeks after tail-vein injection. The data are shown as the mean ± s.d. of five mice per group. **P < 0.01 as determined by *t*-test.

To determine the mechanisms by which MSK1 expression affects the prognosis of patients with CRC, we examined the results of various public databases. The Kaplan–Meier analysis revealed that patients with CRC with high MSK1 expression had significantly lower survival rates than those with low MSK1 expression (Fig. 1a). As observed in a previous study^39^, patients with high MSK1 expression in breast cancer had a higher survival rate than those with low MSK1 expression (Supplementary Fig. 1a). However, no significant correlation was observed between survival rate and MSK1 expression in patients with other carcinomas (Supplementary Fig. 1b–e). MSK1 expression was higher in patients with advanced-stage CRC (stages III and IV) than in those with early-stage CRC (stages I and II) (Fig. 1b). MSK1 expression was also higher in patients with metastases than in those without (Fig. 1c). Overall, these findings indicate that MSK1 expression is highly correlated with survival rate in patients with CRC, especially in terms of metastasis.

MSK1 expression was higher in mesenchymal cells than in epithelial cells in various CRC cell lines (Fig. 1d, e). To investigate the effect of MSK1 expression on various phenotypes of CRC cells, MSK1 was overexpressed in SW480 cells, where the endogenous MSK1 expression level is inherently low. Notably, the morphology of MSK1-expressing cells differed from that of the control cells—SW480 cells overexpressing MSK1 transformed into mesenchymal cells (Fig. 1f). These findings prompted us to examine the expression levels of EMT marker genes in these cells. Concordantly, MSK1 expression inhibited the expression of epithelial marker proteins (including E-cadherin, occludin and claudin) while promoting the expression of mesenchymal marker proteins (including fibronectin, N-cadherin and vimentin) in SW480 cells (Fig. 1g). In particular, Snail protein levels increased among various proteins belonging to the Snail family, which are regarded as master transcription factors that regulate the EMT process (Fig. 1g). The GSE database analysis confirmed that elevated MSK1 expression resulted in decreased expression of epithelial cell marker genes and increased mesenchymal cell marker genes in patients with CRC (Fig. 1h). MSK1-overexpressing SW480 cells exhibited a minimal difference in proliferation ability compared with that in control cells (Fig. 1i) but significantly increased in vitro migration (Fig. 1j) and invasion abilities (Fig. 1k). Furthermore, after injecting the MSK1-overexpressing SW480 cells or control cells through the tail vein of nude mice, the MSK1-overexpressing SW480 cells significantly increased the metastasis to the lungs compared with that in the control cells (Fig. 1l and Supplementary Fig. 2). SW480 cells are known to be a suitable model for confirming lung nodules via tail vein injection^42,43^. These findings collectively indicate that MSK1 overexpression can induce EMT and metastasis in CRC cells.

### MSK1 increases Snail protein stability by suppressing ubiquitination-dependent Snail degradation

**Fig. 2:**
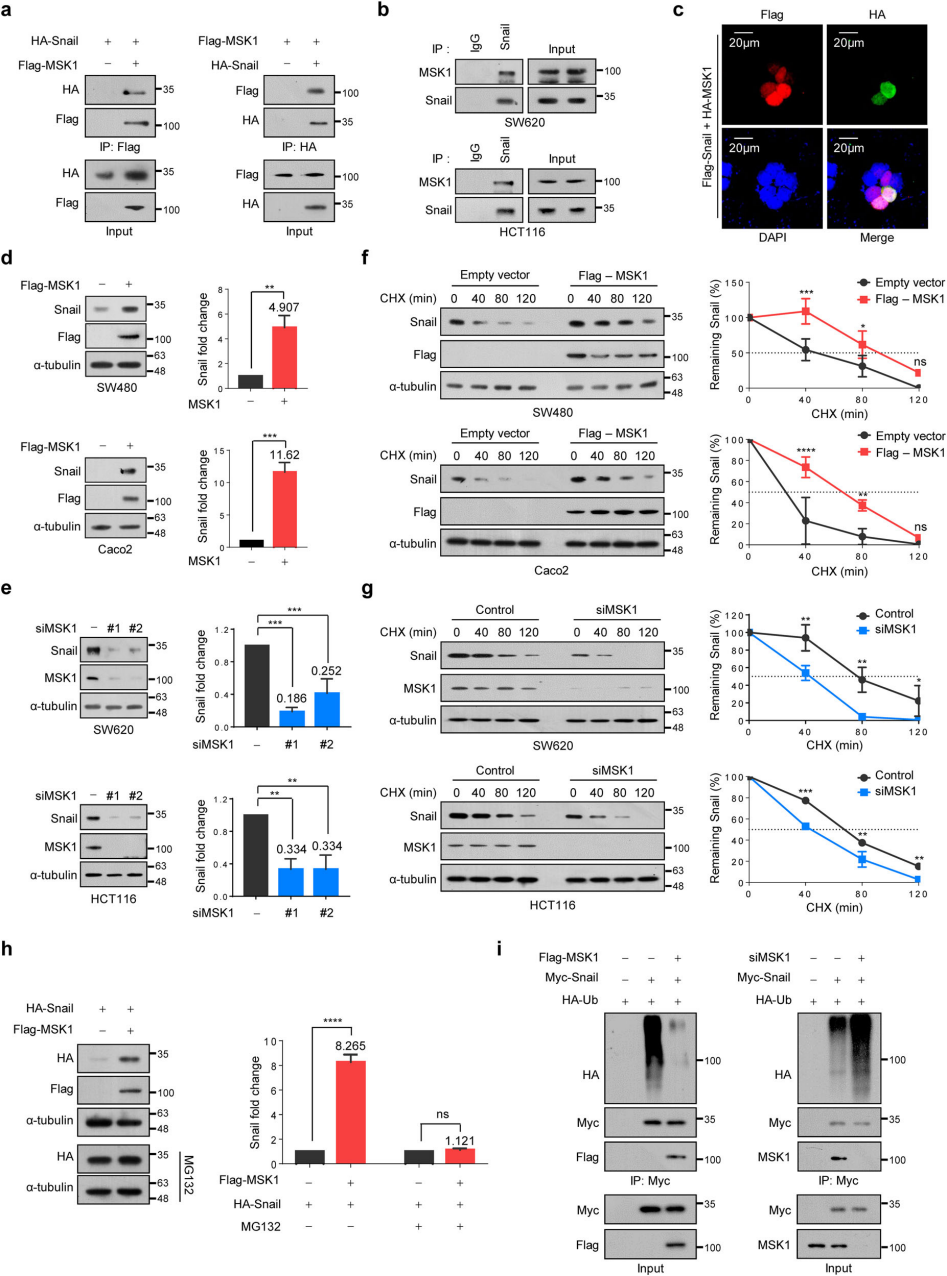
MSK1 increases Snail protein stability by suppressing ubiquitination-dependent Snail degradation. **a** The interaction between exogenous MSK1 and Snail. HA–Snail and Flag–MSK1 were transfected into HEK293T cells. Cell lysates were immunoprecipitated with an anti-Flag or anti-HA and analyzed by western blot using anti-HA (left) or anti-Flag (right) antibody, respectively. **b** The interaction between endogenous MSK1 and Snail. SW620 and HCT116 cell lysates were immunoprecipitated with an anti-Snail and analyzed by western blot using anti-MSK1 antibody. **c** The subcellular localization of MSK1 and Snail. HEK293T cells co-transfected with HA–MSK1 and Flag–Snail were examined by confocal microscopy. Superimposing the three colors (Merge) resulted in pink signals, indicating colocalization of the two proteins in the nucleus. Scale bars, 20 µm. **d** MSK1 was transfected into SW480 or Caco2 cells. Left: cell lysates were immunoblotted with the indicated antibodies. Right: the data are representative of three independent experiments and relative Snail levels were quantified using ImageJ software. **P < 0.01, ***P < 0.001 as determined by *t*-test. **e** Left: immunoblot analysis in MSK1-depleted SW620 or HCT116 cells. Right: the data are representative of three independent experiments, and relative Snail levels were quantified using ImageJ software. **P < 0.01, ***P < 0.001 as determined by *t*-test. **f** MSK1 was transfected into SW480 or Caco2 cells in the presence of CHX (100 µg/ml) for the indicated times. Left: cell lysates were immunoblotted by antibodies as indicated. Right: the data were quantified using ImageJ software. For normalization, α-tubulin expression was used as a control. *P < 0.05, **P < 0.01, ***P < 0.001 as determined by *t*-test. **g** MSK1-depleted SW620 or HCT116 cells were treated with CHX (100 µg/ml) for the indicated times before collection. Left: cell lysates were immunoblotted by antibodies as indicated. Right: the data were quantified using ImageJ software. For normalization, α-tubulin expression was used as a control. *P < 0.05, **P < 0.01, ***P < 0.001 as determined by *t*-test. **h** Left: HA–Snail was co-transfected with Flag–MSK1 into HEK293T cells (top) and then treated with 10 μM MG132 for 12 h (bottom). Cell lysates were immunoblotted with the indicated antibodies. Right: the data are representative of three independent experiments, and relative Snail levels were quantified using ImageJ software. ****P < 0.0001 as determined by *t*-test. **i** MSK1 was either overexpressed (left) or inhibited (right), and Myc–Snail was co-transfected with a plasmid expressing HA–ubiquitin as indicated in HEK293T cells, and then the cells were treated with 10 μM MG132 for 6 h. Cell lysates were immunoprecipitated using an anti-Myc antibody and then analyzed by immunoblotting using an anti-HA tag antibody.

MSK1 increased Snail protein expression in CRC cells (Fig. 1g); therefore, we determined whether MSK1 could affect Snail protein stability. First, we validated the interaction between MSK1 and Snail using reciprocal IP in HEK293T cells co-transfected with vectors encoding HA–Snail and/or Flag–MSK1 (Fig. 2a). Next, we validated the interaction between endogenous MSK1 and Snail in SW620 and HCT116 CRC cells, both of which express high levels of MSK1 (Fig. 2b). Confocal microscopy results revealed that MSK1 and Snail colocalized within the nucleus of HEK293T cells and Snail expression increased further in MSK1-overexpressing cells (Fig. 2c). To determine the Snail domain required for MSK1 interaction, five truncated forms of Snail (Supplementary Fig. 3a) were co-expressed with full-length MSK1, followed by IP using an anti-Flag antibody. All the constructs, excluding the zinc-finger domain-deleted construct (Snail-∆ZnF 1–4), interacted with MSK1 (Supplementary Fig. 3b). We confirmed that Snail interacts with MSK1 in the nucleus (Fig. 2c). However, as the Snail mutant lacking the zinc-finger domain has been reported to be localized exclusively in the cytoplasm^44^, we investigated the interaction between WT-Snail and Snail-∆ZnF 1–4 with recombinant MSK1 in vitro after expressing them individually in cells. The results revealed that WT-Snail binds to MSK1, whereas Snail-∆ZnF 1–4 does not (Supplementary Fig. 3c). These findings indicate that the zinc-finger domain of Snail is essential for its interaction with MSK1. MSK1 overexpression significantly increased endogenous Snail protein levels in SW480 and Caco2 cells (Fig. 2d) while having no effect on mRNA-expression levels (Supplementary Fig. 4a). RNAi-mediated MSK1 depletion decreased endogenous Snail protein levels in SW620 and HCT116 cells (Fig. 2e), which have high MSK1 expression, without affecting Snail mRNA levels (Supplementary Fig. 4b). A CHX pulse-chase analysis revealed that MSK1 overexpression significantly increased the half-life of the Snail protein in SW480 and Caco2 cells (Fig. 2f). By contrast, decreased MSK1 expression significantly decreased the half-life of the Snail protein in SW620 and HCT116 cells (Fig. 2g). Collectively, these findings indicate that MSK1 may increase Snail protein stability in CRC cells.

MSK1 also increased the level of exogenously expressed Snail in HEK293T cells (Fig. 2h). Notably, inhibiting proteasome function with MG132 significantly increased exogenous Snail expression to the same level irrespective of MSK1 expression (Fig. 2h), suggesting that MSK1 may increase Snail protein stability by inhibiting proteasome-dependent Snail degradation. We also observed that MSK1 expression significantly decreased Snail ubiquitination (Fig. 2i, left). By contrast, the ubiquitination increased upon MSK1 depletion (Fig. 2i, right). Overall, these findings suggest that MSK1 may increase Snail protein stability by suppressing ubiquitination-dependent Snail degradation.

### MSK1 kinase activity is essential to increase the stability of Snail protein

**Fig. 3:**
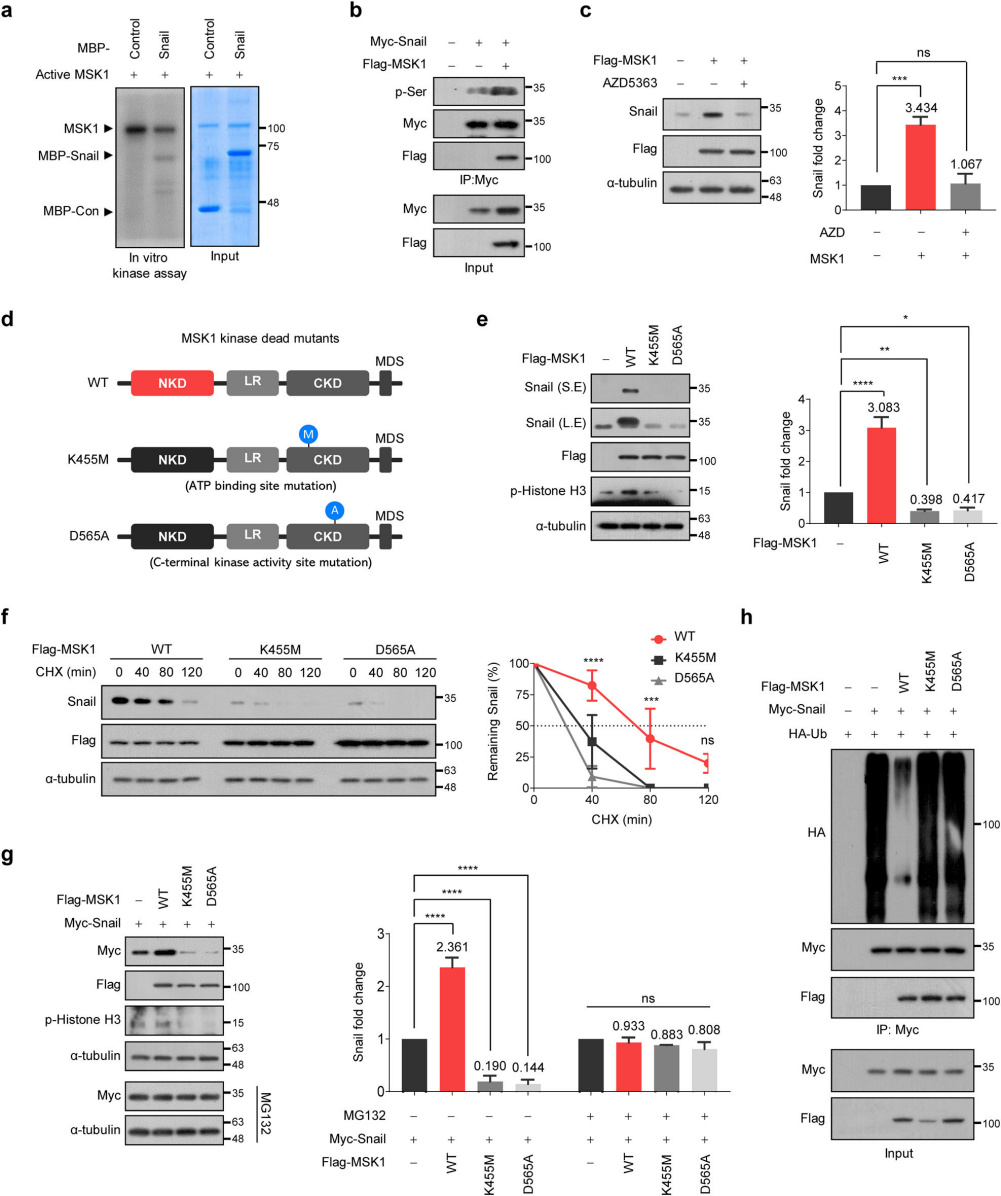
MSK1 kinase activity is essential to increase the stability of Snail protein. **a** In vitro kinase assays were performed by incubating recombinant active MSK1 protein with purified MBP-fused Snail in the presence of [γ-^32^P]-ATP. The resulting products were subjected to SDS–polyacrylamide gel electrophoresis and autoradiography. **b** Myc–Snail was co-transfected with Flag–MSK1 into HEK293T cells, and then the cells were treated with 10 μM MG132 for 6 h. Cell lysates were immunoprecipitated using an anti-Myc antibody and then analyzed by immunoblotting using an anti-phospho-Ser (p-Ser) antibody. **c** MSK1 was transfected into HEK293T cells, and then the cells were treated with 1 μM AZD5363 for 24 h. Left: cell lysates were immunoblotted with the indicated antibodies. Right: the data are representative of three independent experiments and relative Snail levels were quantified using ImageJ software. ***P < 0.001 as determined by *t*-test. **d** A schematic diagram showing the functional domains of MSK1 and mutation sites for kinase-dead MSK1 mutant constructs (K455M and D565A). **e** WT-MSK1, K455M-MSK1 or D565A-MSK1 was transfected into HEK293T cells. Left: cell lysates were immunoblotted with the indicated antibodies. Histone is one of the MSK1 substrate. S.E., short exposure; L.E., long exposure. Right: the data are representative of three independent experiments, and relative Snail levels were quantified using ImageJ software. *P < 0.05, **P < 0.01, ****P < 0.0001 as determined by *t*-test. **f** WT-MSK1, K455M-MSK1 or D565A-MSK1 was *t*ransfected into HEK293T cells in the presence of CHX (100 µg/ml) for the indicated times. Left: cell lysates were immunoblotted by antibodies as indicated. Right: the data were quantified using ImageJ software. For normalization, α-tubulin expression was used as a control. ***P < 0.001, ****P < 0.0001 as determined by *t*-test. **g** Left: HA–Snail was co-transfected with WT-MSK1, K455M-MSK1 or D565A-MSK1 into HEK293T cells (top) and then treated with 10 μM MG132 for 12 h (bottom). Cell lysates were immunoblotted with the indicated antibodies. Right: the data are representative of three independent experiments, and relative Snail levels were quantified using ImageJ software. ****P < 0.0001 as determined by *t*-test. **h** WT-MSK1, K455M-MSK1 or D565A-MSK1 was co-transfected with plasmids expressing Myc–Snail and HA–ubiquitin as indicated in HEK293T cells, and then the cells were treated with 10 μM MG132 for 6 h. Cell lysates were immunoprecipitated using an anti-Myc antibody and then analyzed by immunoblotting using an anti-HA tag antibody.

The phosphorylation status of Snail is crucial for regulating its stability^17,20,21,24,25,45–51^; therefore, we next investigated whether Snail could be a direct substrate of MSK1. We first performed in vitro kinase assays using commercially available recombinant active MSK1 and purified MBP-fused Snail proteins and observed that Snail was phosphorylated by active MSK1 in vitro (Fig. 3a and Supplementary Fig. 5). To confirm MSK1-mediated Snail phosphorylation in cells, we co-transfected Snail with MSK1 and observed that MSK1 phosphorylated Snail protein in HEK293T cells (Fig. 3b). To determine which serine residue could be phosphorylated by MSK1, we substituted serine residues in Snail predicted to be phosphorylated by MSK1 with alanine. Despite these substitutions, all mutants were still phosphorylated by MSK1, indicating that multiple serine residues in Snail are probably phosphorylated by MSK1 (Supplementary Fig. 6). To determine whether the kinase activity of MSK1 is required to increase Snail protein expression, we treated HEK293T, SW620 and HCT116 cells with AZD5363, an inhibitor of MSK1. The MSK1-induced increase in Snail protein was no longer observed in AZD5363-treated HEK293T cells (Fig. 3c). In addition, AZD5363 treatment significantly decreased Snail expression in SW620 and HCT116 cells expressing high MSK1 levels (Supplementary Fig. 7). To more directly validate whether the kinase activity of MSK1 is essential for increasing the stability of Snail proteins, we generated two MSK1 mutant constructs (K455M and D565A) that lack kinase activity (Fig. 3d)^26,52,53^. We observed that these two mutants interacted with Snail similarly to WT MSK1 (Supplementary Fig. 8a) but could not phosphorylate Snail protein (Supplementary Fig. 8b). Overexpression of K455M-MSK1 or D565A-MSK1 in HEK293T, SW480 or Caco2 cells did not increase the expression (Fig. 3e and Supplementary Fig. 9a) or stability (Fig. 3f and Supplementary Fig. 9b) of Snail proteins. In addition, when these mutants were expressed in HEK293T cells, unlike WT MSK1, the exogenously expressed Snail protein levels were not increased but decreased (Fig. 3g), indicating that the expression of these mutants could suppress the function of endogenously expressed MSK1. Snail protein expression levels were comparable under all conditions when MG132 was treated (Fig. 3g). Furthermore, the expression of WT MSK1 decreased Snail protein ubiquitination. However, the expression of these mutants did not decrease Snail protein ubiquitination (Fig. 3h). These findings indicate that the kinase activity of MSK1 is essential to increase Snail protein stability by inhibiting its ubiquitination.

### MSK1 kinase activity is essential to promote CRC cell metastasis

**Fig. 4:**
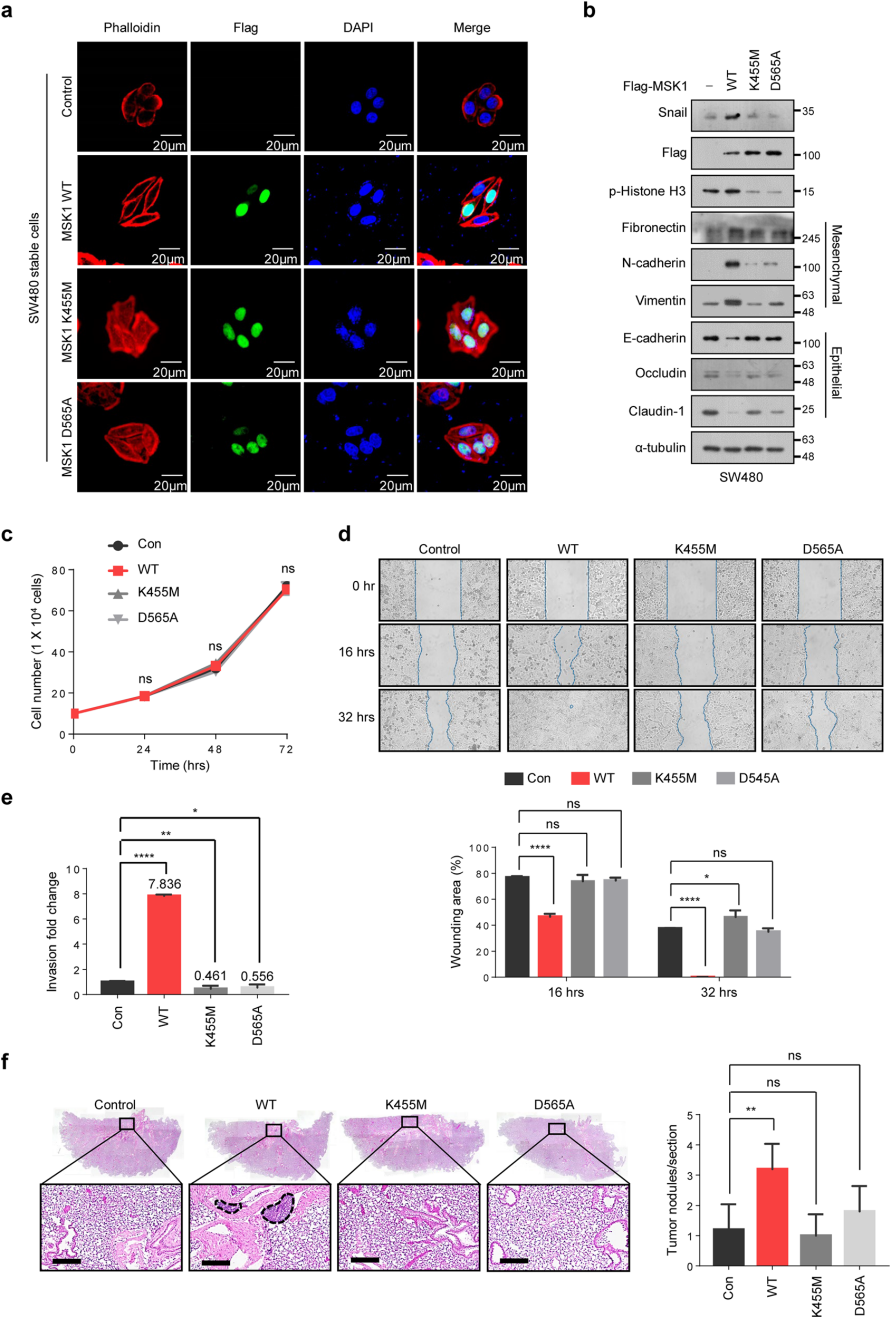
MSK1 kinase activity is essential to promote CRC cell metastasis. **a** Morphological changes of WT-MSK1-, K455M-MSK1- or D565A-MSK1-expressing SW480 cells. MSK1-overexpressing SW480 and control cells visualized by confocal microscopy after staining with TRITC-conjugated phalloidin. Scale bars, 20 µm. **b** Expression levels of EMT marker proteins in WT-MSK1-, K455M-MSK1- or D565A-MSK1-expressing SW480 and control cells were analyzed by immunoblotting. **c** The indicated cells were seeded in a six-well plate at a concentration of 1 × 10^5^ cells per well. After incubation for 1–4 days, the viable cells were counted with a hemocytometer after trypan blue staining**. d** Left: WT-MSK1-, K455M-MSK1- or D565A-MSK1-expressing SW480 and control cells were analyzed in wound-healing assays by visualizing wound closure via phase-contrast microcopy. Wound areas were measured using WimScratch software (Wimasis). Right: the data shown represent the percentage of the wound area and are expressed as the mean ± s.d. of three individual experiments. *P < 0.05, ****P < 0.0001 as determined by *t*-test. **e** WT-MSK1-, K455M-MSK1- or D565A-MSK1-expressing SW480 and control cells were seeded onto Matrigel matrix-coated top chambers, and the fold changes of invading cells were measured after 30 h. The data shown are expressed as the mean ± s.d. of three individual experiments, each performed in triplicate. *P < 0.05, **P < 0.01, ****P < 0.0001 as determined by *t*-test. **f** About 2 × 10^6^ of the SW480 cells stably overexpressing WT-MSK1, K455M-MSK1, D565A-MSK1 or control cells were injected into the nude mice by tail-vein injection. Left: representative pictures of HE staining of lung sections. Scale bars, 100 μm. Right: the number of metastatic lung nodules in individual mice was quantified at 10 weeks after tail-vein injection. The data are shown as the mean ± s.d. of five mice per group. **P < 0.01 as determined by *t*-test.

To investigate the role of MSK1 kinase activity in CRC cell metastasis, we developed stable cell lines expressing either K455M-MSK1 or D565A-MSK1 in SW480 cells and compared the metastatic phenotypes with those of WT-MSK1-expressing SW480 cells. Unlike SW480 cells overexpressing WT MSK1, mutant MSK1 (K455M or D565A)-expressing SW480 cells maintained epithelial shape (Fig. 4a), and little change in the expression of various EMT marker proteins was observed (Fig. 4b). SW480 cells overexpressing mutant MSK1 (K455M or D565A) did not proliferate any differently than control cells, nor did WT MSK1-expressing SW480 cells (Fig. 4c). Unlike SW480 cells overexpressing WT MSK1, mutant MSK1 (K455M or D565A)-expressing SW480 cells did not increase their in vitro migration (Fig. 4d) and invasion (Fig. 4e) abilities, as well as their in vivo metastatic abilities (Fig. 4f and Supplementary Fig. 10), compared with control cells. These findings imply that the kinase activity of MSK1 is essential to promote CRC cell metastasis.

### Snail is essential for MSK1-induced EMT and an increase in CRC cell metastatic ability

**Fig. 5:**
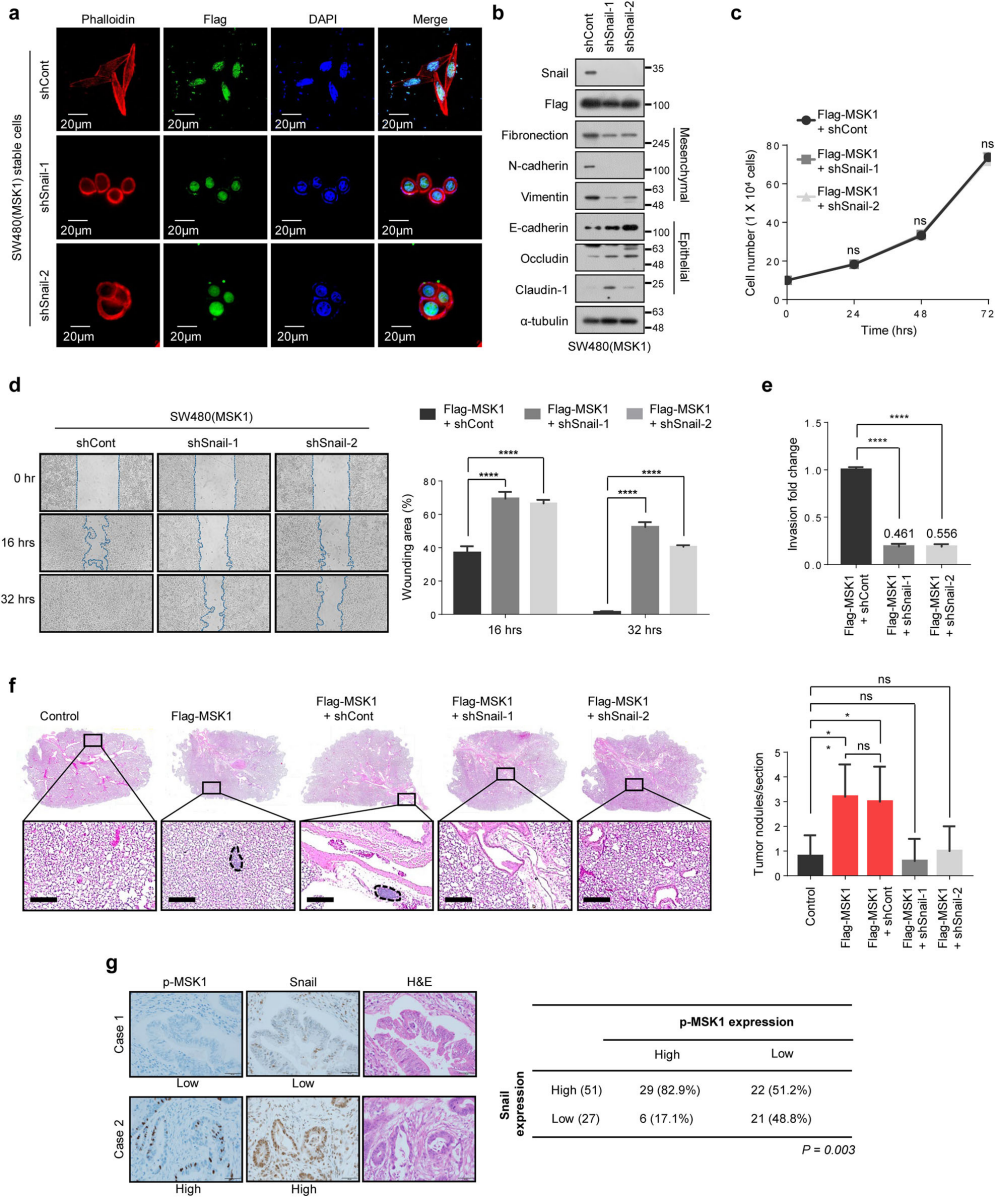
Snail is essential for MSK1-induced EMT and an increase in CRC cell metastatic ability. **a** Morphological changes of Snail-depleted MSK1-overexpressing SW480 cells. Snail-depleted MSK1-overexpressing SW480 and control cells visualized by confocal microscopy after staining with TRITC-conjugated phalloidin. Scale bars, 20 µm. **b** Expression levels of EMT marker proteins in Snail-depleted MSK1-overexpressing SW480 and control cells were analyzed by immunoblotting. **c** The indicated cells were seeded in a six-well plate at a concentration of 1 × 10^5^ cells per well. After incubation for 1–4 days, the viable cells were counted with a hemocytometer after trypan blue staining**. d** Left: Snail-depleted MSK1-overexpressing SW480 and control cells were analyzed in wound-healing assays by visualizing wound closure via phase-contrast microcopy. Wound areas were measured using WimScratch software (Wimasis). Right: the data shown represent the percentage of the wound area and are expressed as the mean ¡± s.d. of three individual experiments. ****P < 0.0001 as determined by *t*-test. **e** Snail-depleted MSK1-overexpressing SW480 and control cells were seeded onto Matrigel matrix-coated top chambers, and the fold changes of invading cells were measured after 30 h. The data shown are expressed as the mean ± s.d. of three individual experiments, each performed in triplicate. ****P < 0.0001 as determined by *t*-test. **f** About 2 × 10^6^ of the Snail-depleted MSK1-overexpressing SW480 or control cells were injected into the nude mice by tail-vein injection. Left: representative pictures of H&E staining of lung sections. Scale bars, 100 μm. Right: the number of metastatic lung nodules in individual mice was quantified at 10 weeks after tail-vein injection. The data are shown as the mean ± s.d. of five mice per group. *P < 0.05 as determined by *t*-test. **g** Left: representative IHC images of phospho-MSK1 and Snail in colorectal tumors. Scale bars, 50 μm. Left: correlation of the Snail expression levels with the phospho-MSK1 levels, as determined using a colorectal tumor TMA. Statistical significances were determined by Pearson’s *χ*^2^ test and Fisher’s exact test.

To determine whether Snail is an important mediator of EMT in MSK1-overexpressing SW480 cells, lentiviral shRNA (shSnail-1 and shSnail-2) was used to downregulate Snail expression. Snail depletion reverted the EMT-associated morphological changes induced by MSK1 expression (Fig. 5a). In addition, Snail depletion reversed the downregulation of epithelial marker proteins (including E-cadherin, occludin and claudin) and upregulation of mesenchymal marker proteins (including fibronectin, N-cadherin and vimentin) (Fig. 5b), suggesting that increased Snail expression is critical for the induction of EMT in MSK1-overexpressing SW480 cells. In a subsequent evaluation to determine whether Snail depletion could alter the metastatic ability of MSK1-overexpressing SW480 cells, we found that Snail depletion did not affect cellular growth rates (Fig. 5c) but significantly suppressed in vitro migration (Fig. 5d) and invasion (Fig. 5e) abilities, as well as the in vivo metastatic abilities (Fig. 5f and Supplementary Fig. 11) of these cells. These results indicate that the upregulation of Snail expression plays a key role in MSK1-induced CRC cell metastasis.

To determine whether MSK1 activation plays a significant role in increasing Snail expression in patients with CRC, we performed TMA analysis on 78 CRC tissue specimens. We observed a strong positive correlation between MSK1 activity (that is, the phospho-MSK1 level) and Snail protein expression (Fig. 5g). In addition, an analysis of a public microarray dataset (GSE68468) of metastatic colorectal tumor samples revealed that MSK1 expression had a negative correlation with E-cadherin expression and a positive correlation with N-cadherin expression (Supplementary Fig. 12). Our findings indicate that MSK1 can regulate Snail expression in CRC patients.

### USP5 is essential for the MSK1-induced increase in the stability of Snail protein

**Fig. 6:**
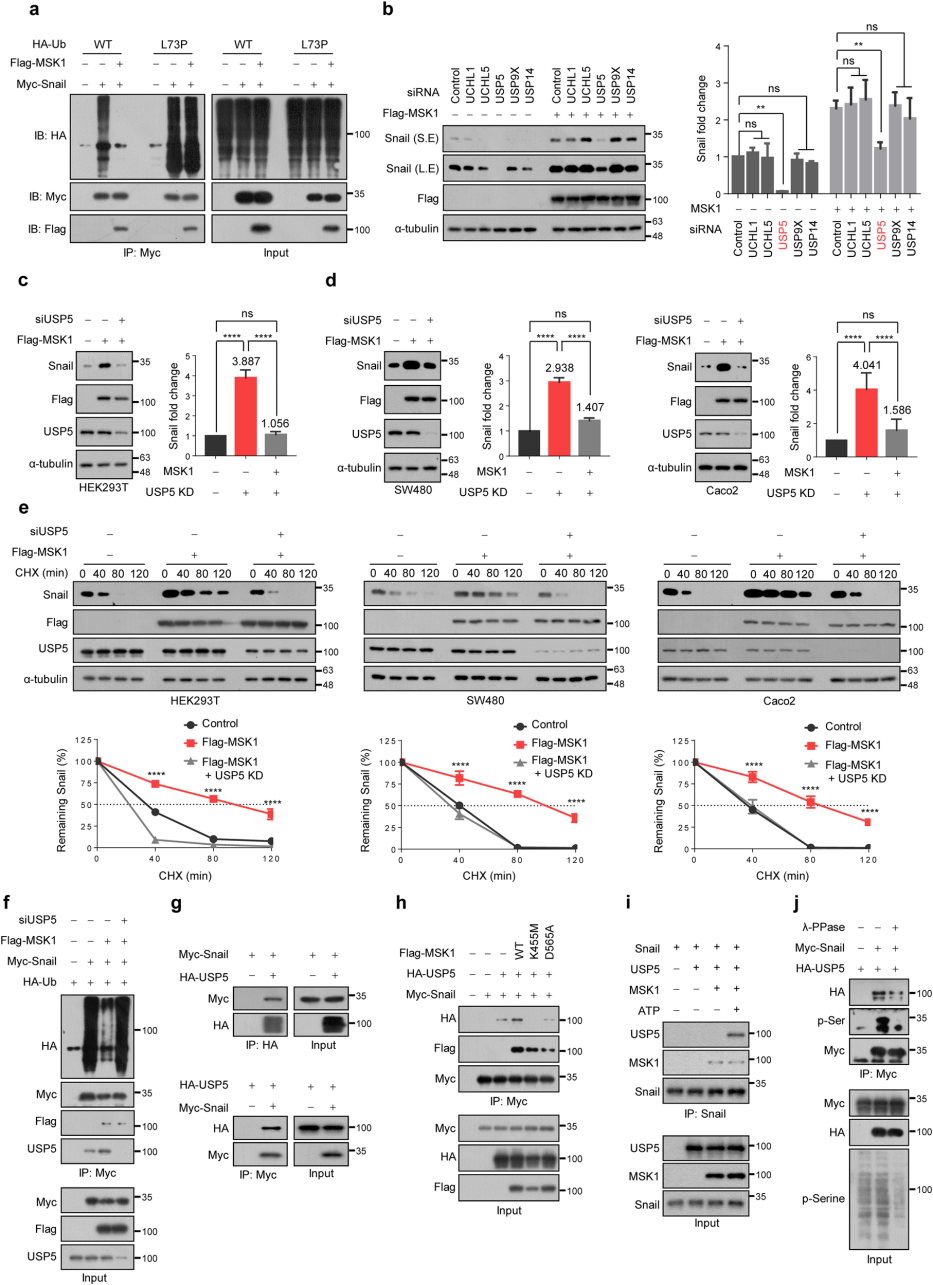
USP5 is essential for the MSK1-induced increase in the stability of Snail protein. **a** HA-tagged WT- or L73P-ubiquitin was co-transfected with plasmids expressing Myc–Snail and Flag–MSK1 as indicated in HEK293T cells, and then the cells were treated with 10 μM MG132 for 6 h. Cell lysates were immunoprecipitated using an anti-Myc antibody and then analyzed by immunoblotting using an anti-HA tag antibody. **b** Left: each of the five DUB-specific siRNAs was co-transfected with MSK1 in HEK293T cells. Cell lysates were immunoblotted with the indicated antibodies. S.E., short exposure; L.E., long exposure. Right: the data are representative of three independent experiments, and relative Snail levels were quantified using ImageJ software. **P < 0.01 as determined by *t*-test. **c** Left: USP5-specific siRNA was co-transfected with MSK1 in HEK293T cells. Cell lysates were immunoblotted with the indicated antibodies. Right: the data are representative of three independent experiments, and relative Snail levels were quantified using ImageJ software. ****P < 0.0001 as determined by *t*-test. **d** USP5-specific siRNA was co-transfected with MSK1 in SW480 or Caco2 cells. Left: cell lysates were immunoblotted with the indicated antibodies. Right: the data are representative of three independent experiments, and relative Snail levels were quantified using ImageJ software. ****P < 0.0001 as determined by *t*-test. **e** USP5-specific siRNA was co-transfected with MSK1 in SW480 or Caco2 cells in the presence of CHX (100 µg/ml) for the indicated times. Left: cell lysates were immunoblotted by antibodies as indicated. Right: the data were quantified using ImageJ software. For normalization, α-tubulin expression was used as a control. ****P < 0.0001 as determined by *t*-test. **f** USP5-specific siRNA was co-transfec*t*ed with plasmids expressing Myc–Snail, Flag–MSK1 and HA–ubiquitin as indicated in HEK293T cells, and then the cells were treated with 10 μM MG132 for 6 h. Cell lysates were immunoprecipitated using an anti-Myc antibody and then analyzed by immunoblotting using an anti-HA tag antibody. **g** The interaction between exogenous USP5 and Snail. Myc–Snail and HA–USP5 were transfected into HEK293T cells. Cell lysates were immunoprecipitated with an anti-HA or anti-Myc and analyzed by western blot using anti-Myc (top) or anti-HA (bottom) antibody, respectively. **h** The interaction between exogenous USP5 and Snail in the presence of WT-MSK1, K455M-MSK1 or D565A-MSK1. Myc–Snail and HA–USP5 were co-transfected with WT-MSK1, K455M-MSK1 or D565A-MSK1 into HEK293T cells and then treated with 10 μM MG132 for 6 h. Cell lysates were immunoprecipitated with an anti-Myc and analyzed by western blot using anti-HA or anti-Flag antibody. **i** The direct interaction between USP5 and Snail in vitro. The kinase reactions were performed by incubating recombinant active MSK1 protein, recombinant Snail protein and recombinant USP5 protein in the kinase buffer with or without the presence of ATP for 1 h at 30 °C. After IP of each sample with anti-Snail antibody, western blot analysis was conducted using anti-USP5 antibody. **j** The interaction between exogenous USP5 and Snail in the presence of λ-phosphatase. Myc–Snail and HA–USP5 were transfected into HEK293T cells. Cell lysates were treated with λ-phosphatase for 30 min at 30 °C, and immunoprecipitated with an anti-Myc and analyzed by western blot using anti-HA antibody.

Because MSK1 decreased Snail ubiquitination, we determined whether MSK1 inhibits ubiquitination or promotes deubiquitination of Snail protein. To that end, we generated an L73P deubiquitinating enzyme (DUB)-resistant ubiquitin mutant^54^ to determine the degree of Snail ubiquitination by MSK1. MSK1 expression decreased the ubiquitination of the Snail protein when WT ubiquitin was used but not when L73P ubiquitin was used, even when MSK1 was expressed (Fig. 6a). These findings indicate that MSK1 does not inhibit Snail protein ubiquitination but rather promotes its deubiquitination, which, in turn, increases Snail protein stability.

Subsequently, we used the DUB inhibitors PR-619 and WP1130 to investigate DUB, which plays a significant role in increasing the stability of Snail protein by MSK1. In HEK293T cells, PR-619 treatment reduced the MSK1-mediated increase in Snail protein levels to a greater extent than in control cells lacking MSK1 expression (Supplementary Fig. 13a, lane 4 versus lane 1). However, the WP1130 treatment only reduced the MSK1-mediated increase in Snail protein levels to the same extent as in control cells lacking MSK1 expression (Supplementary Fig. 13b, lane 4 versus lane 1). Furthermore, PR-619 treatment after MSK1 inhibition more potently decreased Snail protein levels compared with that of MSK1 inhibition alone (Supplementary Fig. 13c, lane 4 versus lane 3); however, no further decrease was observed upon WP1130 treatment (Supplementary Fig. 13d, lane 4 versus lane 3). These findings indicate that PR-619 inhibits DUB action independently of MSK1, but WP1130 inhibits DUB action, which is essential for MSK1 to increase Snail protein expression.

While PR-619 is a general DUB inhibitor that inhibits many DUBs, WP1130 inhibits a subgroup of DUBs, including UCHL1, UCHL5, USP5, USP9X and USP14 (ref. ^55^). To determine which of these five DUBs would affect MSK1-induced Snail protein expression, we knocked down each corresponding gene using siRNA. We observed that USP5 depletion no longer resulted in an increase in MSK1-induced Snail protein expression in HEK293T cells (Fig. 6b, c). In SW480 and Caco2 cells, decreasing USP5 expression significantly decreased Snail expression levels, which were increased by MSK1 expression (Fig. 6d). In addition, MSK1 did not extend the half-life of the Snail protein when USP5 was depleted (Fig. 6e), nor did it reduce Snail protein ubiquitination (Fig. 6f). These findings indicate that USP5 is required for MSK1 to increase Snail protein stability by promoting Snail protein deubiquitination. Because the kinase activity of MSK1 was essential for Snail protein stability (Fig. 3), we assumed that, when Snail protein was phosphorylated by MSK1, the interaction with USP5 would increase. We first confirmed that exogenously expressed USP5 interacts with Snail in HEK293T cells (Fig. 6g) and that the zinc-finger domain of Snail plays an important role in its interaction with USP5 (Supplementary Fig. 14). Furthermore, their interaction increased when WT MSK1 was co-expressed, but their interaction decreased when K455M and D565A mutant MSK1 were co-expressed (Fig. 6h). We also confirmed that, while recombinant Snail and USP5 did not bind in vitro, phosphorylated Snail by MSK1 was able to bind to USP5 (Fig. 6i). Finally, when the Snail protein was dephosphorylated using λ-phosphatase, the USP5–Snail interaction was significantly decreased (Fig. 6j). All of these findings suggest that USP5 promotes Snail protein deubiquitination by binding to MSK1-phosphorylated Snail protein.

### MSK1 regulates Snail protein expression and its interaction with USP5 under physiological conditions

**Fig. 7:**
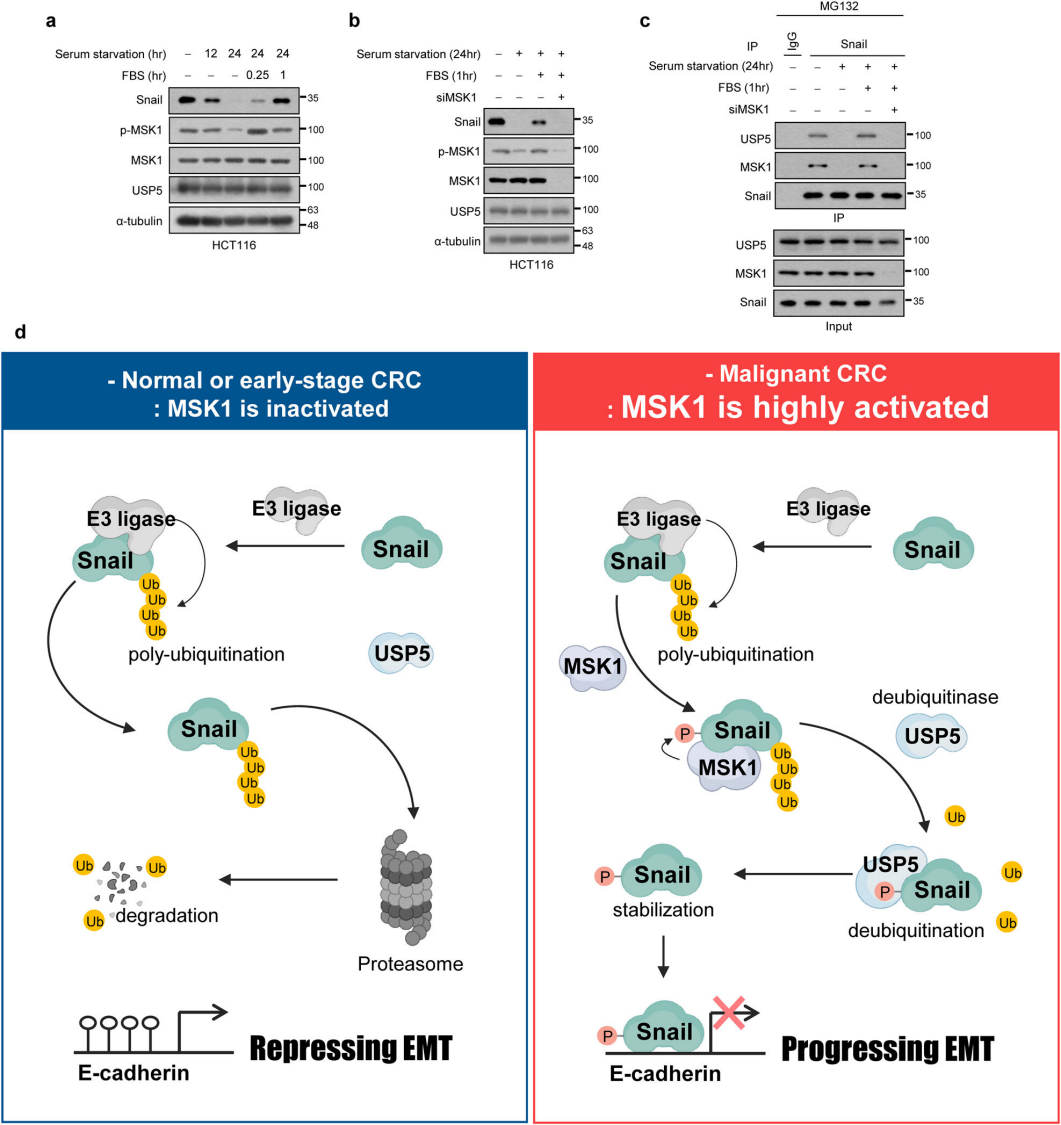
MSK1 regulates Snail protein expression and its interaction with USP5 under physiological conditions. **a** HCT116 cells were incubated for the indicated times in serum-free medium and then stimulated with DMEM medium including 10% FBS. Cell lysates were immunoblotted with the indicated antibodies. **b** MSK1-depleted HCT116 cells were incubated for 24 h in serum-free medium and then stimulated with DMEM medium including 10% FBS for 1 h. Cell lysates were immunoblotted with the indicated antibodies. **c** Serum-induced interaction between Snail and USP5. MSK1-depleted HCT116 cells were incubated for 24 h in serum-free medium and then stimulated with DMEM medium including 10% FBS for 1 h. The cells were treated with 10 μM MG132 for 6 h before collection. Cell lysates were immunoprecipitated with an anti-Snail and analyzed by western blot using anti-USP5 or anti-MSK1 antibody. The data are representative of three independent experiments. **d** A schematic diagram showing how stabilization of Snail by MSK1 leads to EMT in CRC. In normal or early-stage CRC cells, MSK1 is inactivated (or the expression of MSK1 is low) and Snail is readily degraded through UPS, thereby suppressing EMT. In malignant CRC cells, MSK1 is highly activated, and activated MSK1 can increase Snail protein stability by enhancing USP5-mediated Snail deubiquitination, with the resulting high Snail expression being able to promote EMT and tumor metastasis.

To investigate the role of MSK1 in the interaction between Snail and USP5 under physiological conditions, we induced serum starvation in HCT116 cells, which have high expression of MSK1 (Fig. 1e) and confirmed interaction of MSK1 with Snail (Fig. 2b). We observed a decrease in both MSK1 activity and Snail protein expression upon serum starvation (Fig. 7a). Upon reintroduction of serum, both MSK1 activity and Snail protein expression increased (Fig. 7a). However, the mRNA level of Snail did not change significantly under these conditions (Supplementary Fig. 15), suggesting that the regulatory effect of serum on Snail occurs primarily at the level of protein stability rather than transcription. To ascertain the necessity of MSK1 activation for the serum-induced increase in Snail protein expression, we inhibited MSK1 expression. Our findings revealed that inhibiting MSK1 expression prevented the serum-induced increase in Snail protein expression (Fig. 7b), but Snail mRNA levels were not (Supplementary Fig. 15). Furthermore, we confirmed that the interaction between Snail and USP5, which decreased during serum starvation, was restored upon serum treatment (Fig. 7c). However, inhibiting MSK1 expression prevented the restoration of the Snail–USP5 interaction even after serum treatment (Fig. 7c). These results suggest that MSK1 mediates the increase in Snail protein expression under physiological conditions and facilitates the interaction between phosphorylated Snail protein and USP5.

## Discussion

MSK1 is closely associated with CRC metastasis; however, its mechanism of action remained unknown. In this study, we revealed the precise molecular mechanism by which MSK1 increases the stability of Snail protein to induce EMT and promote CRC metastasis. Our results suggest a model where MSK1 is inactivated (or the expression of MSK1 is low) in normal or early-stage CRC cells, where Snail could be readily degraded through the ubiquitination–proteasome system (UPS), thereby suppressing EMT. By contrast, MSK1 is highly activated in malignant CRC cells, and activated MSK1 can increase Snail protein stability by enhancing USP5-mediated Snail deubiquitination, with the resulting high Snail expression being able to promote EMT and tumor metastasis (Fig. 7d).

As in the case of other signaling proteins, the activities of transcription factors are regulated by phosphorylation in response to various cellular signals. In the case of Snail, phosphorylation regulates protein stability and function in various ways. The stability of the Snail protein is negatively regulated by ubiquitination-dependent proteasomal degradation in the cytoplasm. Therefore, its stability could be increased by promoting its delivery into the nucleus. p21 (RAC1) activated kinase 1 (PAK1) phosphorylates Snail at Ser246 in the zinc-finger domain, which is critical for its nuclear localization^56^, reportedly increasing protein expression and function^20^. Although the protein machinery involved in nuclear Snail import is unknown, Snail phosphorylation by PAK1 probably increases Snail interaction with the protein involved in nuclear trafficking. MSK1 colocalized with Snail in the nucleus (Fig. 2c), and the intracellular localization of Snail protein remained unaltered with MSK1 overexpression (data not shown); therefore, we concluded that MSK1 did not increase stability by transferring Snail protein from the cytoplasm to the nucleus.

Another way to increase Snail stability is to prevent its ubiquitination. Phosphorylation at Ser100 by Ataxia Telangiectasia Mutated (ATM) kinase and DNA-dependent protein kinase catalytic subunit (DNA-PKcs) decreases Snail ubiquitination or its interaction with GSK3β, both of which are critical for its degradation, thereby increasing its stability^22,23^. Erk2-mediated Snail phosphorylation at Ser82/Ser104 residues also protected it from ubiquitination and subsequent proteasomal degradation^24^. We previously proposed that p38 MAPK increases Snail protein stability by suppressing dual-specificity tyrosine-phosphorylation-regulated kinase 2 (DYRK2)-mediated prime phosphorylation of GSK3β, which is critical for βTrCP-mediated Snail ubiquitination and subsequent degradation^25^. MSK1 promoted Snail deubiquitination rather than inhibiting its ubiquitination (Fig. 6a); therefore, we further aimed to identify a new deubiquitinase involved in this process.

A third way could be to increase Snail deubiquitination. Many deubiquitinases, including DUB3, OTUB1, USP1, USP3, USP11, USP18, USP26, USP37 and USP27X, remove ubiquitination from Snail proteins, thereby increasing its intracellular stability^57–66^. However, no kinase has been implicated in promoting Snail deubiquitination thus far. In this study, we elucidated the molecular mechanism by which MSK1 phosphorylation can promote Snail deubiquitination and increase its stability—MSK1 phosphorylates Snail protein, increasing its interaction with USP5 and, consequently, its deubiquitination. Protein phosphorylation either increases or decreases their binding force to deubiquitinase. For instance, in the case of β-catenin, phosphorylation by mitogen-activated protein kinase kinase kinase 2 (MEKK2) increases its interaction with USP15 (ref. ^67^), and in the case of hypoxia-inducible factor 2-alpha (HIF2A), phosphorylation by Erk increases its interaction with USP33 (ref. ^68^). Conversely, phosphorylation of ubiquitin-like, containing PHD and RING finger domains, 1 (UHRF1) by cyclin-dependent kinase 1 (CDK1) decreases its interaction with USP7 (ref. ^69^).

In this study, we demonstrate that MSK1 can induce EMT and promote CRC metastasis by increasing Snail protein stability through USP5-mediated deubiquitination. Our findings reveal a critical mechanism underlying MSK1-induced cancer metastasis and have significant implications for the development of treatment strategies for metastatic CRC.

## Supplementary information

Supplementary Figs. 1–15
