# Supplementary material for: MSK1 promotes colorectal cancer metastasis by increasing Snail protein stability through USP5-mediated Snail deubiquitination
Source: Exp Mol Med. 2025 Apr 1;57(4):820–35. doi: 10.1038/s12276-025-01433-0 (PMC12046000; doi:10.1038/s12276-025-01433-0)
Supplement: Supplementary file 1 — Supplementary Figs. 1–15 [file 12276_2025_1433_MOESM1_ESM.pdf]

Supplementary Figure 1

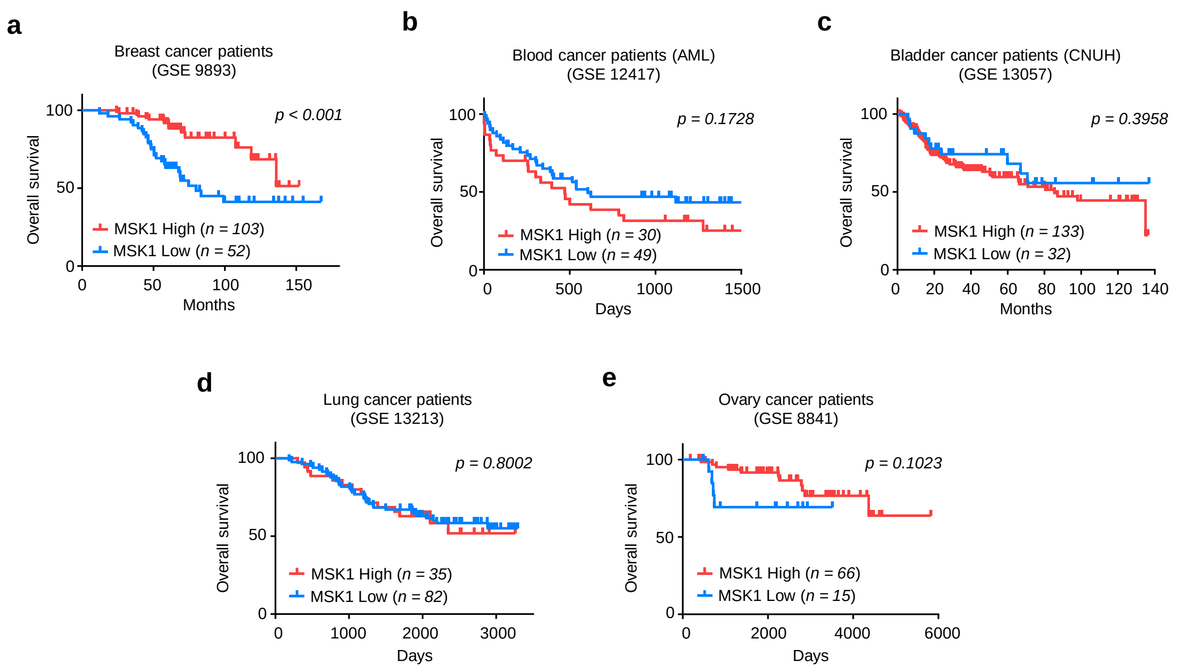

Supplementary Fig. 1. Association of MSK1 expression with overall survival in patients with various cancers.
(a) Kaplan-Meier plot showing the increased survival probability of patients with high levels of MSK1 mRNA (n=103) compared with low MSK1 mRNA levels (n=52) in breast cancer (GSE 9893). Statistical analysis was performed using log-rank tests. (b-e) Kaplan-Meier plot showing no significant correlation between survival rate and MSK1 expression in blood cancer (b), bladder cancer (c), lung cancer (d), and ovary cancer (e).

Supplementary Figure 2

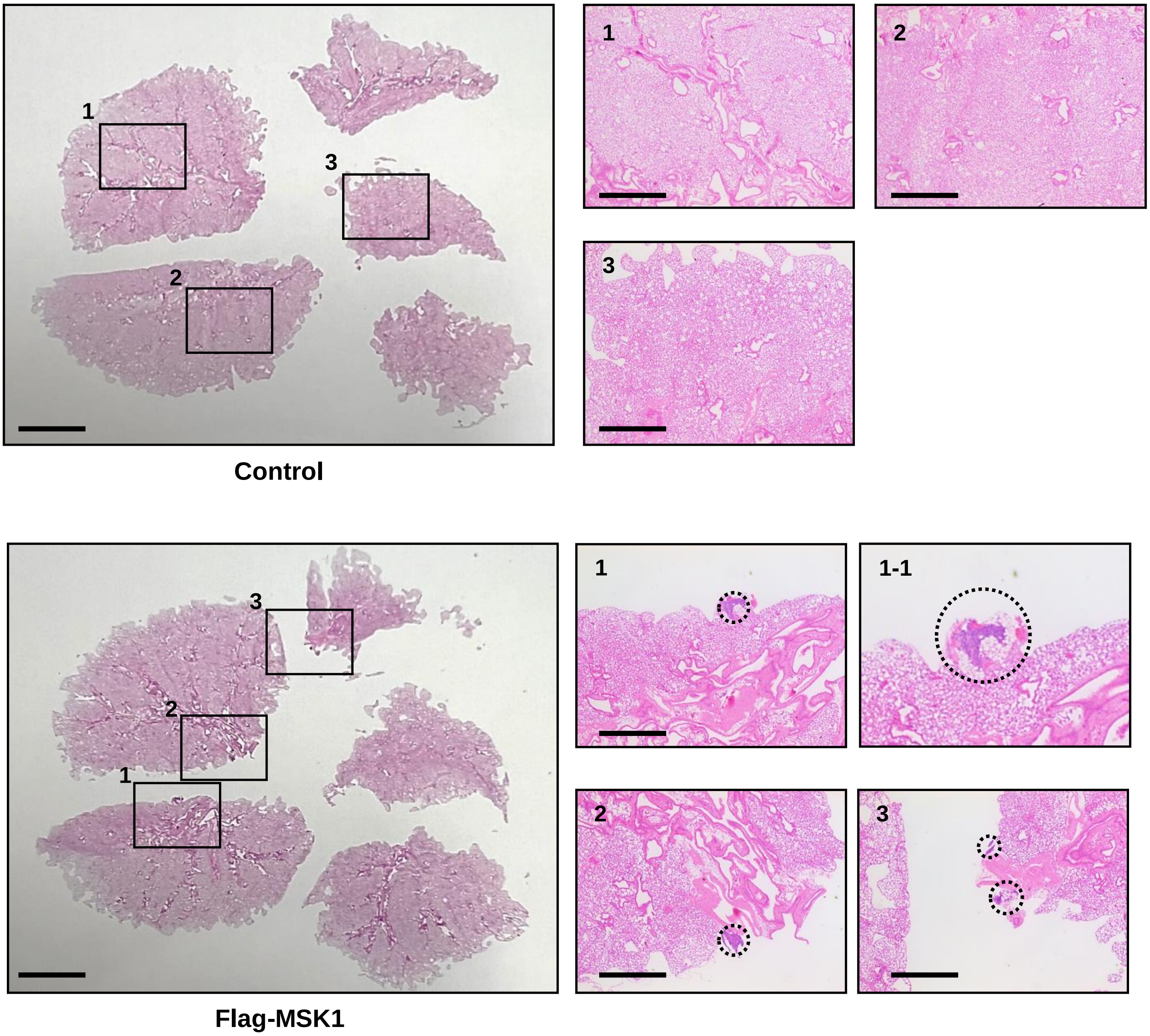

Supplementary Fig. 2. HE staining of whole lung sections after injection of control and MSK1-overexpressing SW480 cells.
Representative HE-stained images of the entire lung 45 days after tail-vein injection of control and MSK1-overexpressing SW480 cells in male BALB/c/nude mice. The images illustrate the histological features and differences between the two conditions. Scale bars in the whole lung: 3 mm, Scale bars in the magnified image: 100 μm.

Supplementary Figure 3

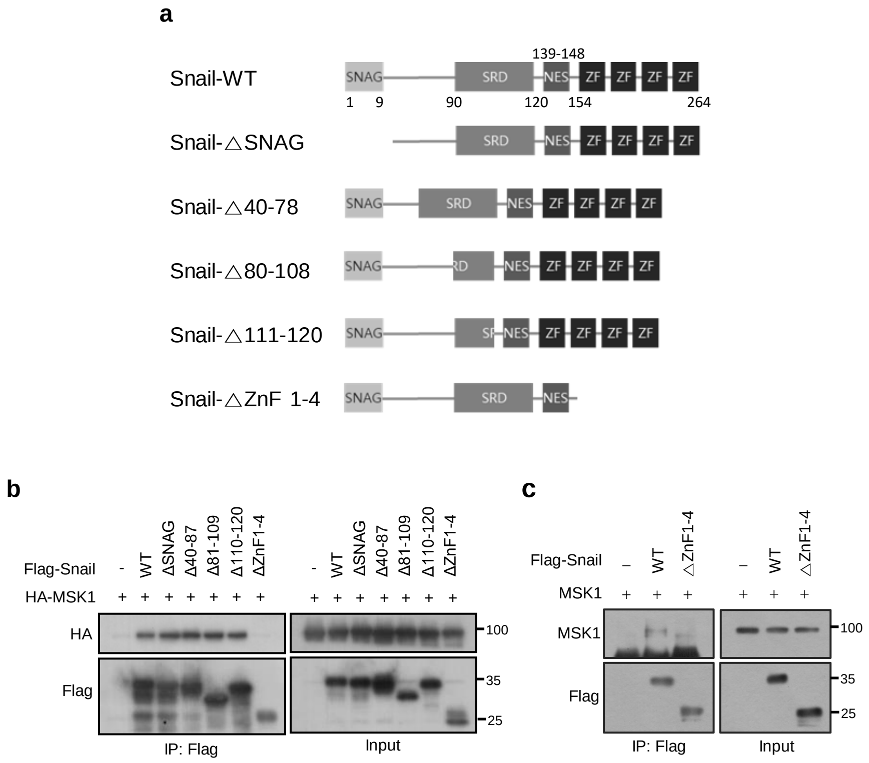

Supplementary Fig. 3. The zinc finger domain of Snail is essential for its interaction with MSK1. (a) Schematic diagram for Snail deletion mutants. SNAG, SNAG domain: SRD, Serine-rich domain: NES, Nuclear export sequence: ZF, Zinc Finger domain. (b) Interaction between exogenous MSK1 and Snail deletion mutants. HEK293T cells transfected with HA-MSK1 and Flag-tagged respective Snail deletion mutants were immunoprecipitation with anti-Flag antibody and analyzed by western blot using anti-HA antibody. (c) *In vitro* interaction of MSK1 with WT-Snail or Snail-ΔZnF 1-4. Recombinant MSK1 was mixed with lysates from HEK293T cells transfected with Flag-tagged WT-Snail or Snail-ΔZnF 1-4, and the mixture was incubated at 30℃ for 30 min. Immunoprecipitation was then performed using an anti-Flag and analyzed by western blot using anti-MSK1 antibody.

Supplementary Figure 4

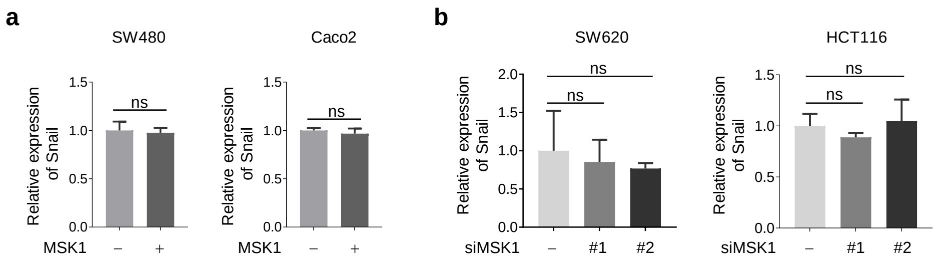

Supplementary Fig. 4. mRNA levels of Snail in MSK1-overexpressing or MSK1-depleted CRC cells. (a) MSK1 was transfected into SW480 or Caco2 cells. mRNA was extracted from each cell lysate and mRNA level of Snail was analyzed by qRT-PCR. For normalization, GAPDH expression was used as a control. (b) MSK1-specific siRNAs were transfected into SW620 or HCT116 cells. mRNA was extracted from each cell lysate and mRNA level of Snail was analyzed by qRT-PCR. For normalization, GAPDH expression was used as a control.

Supplementary Figure 5

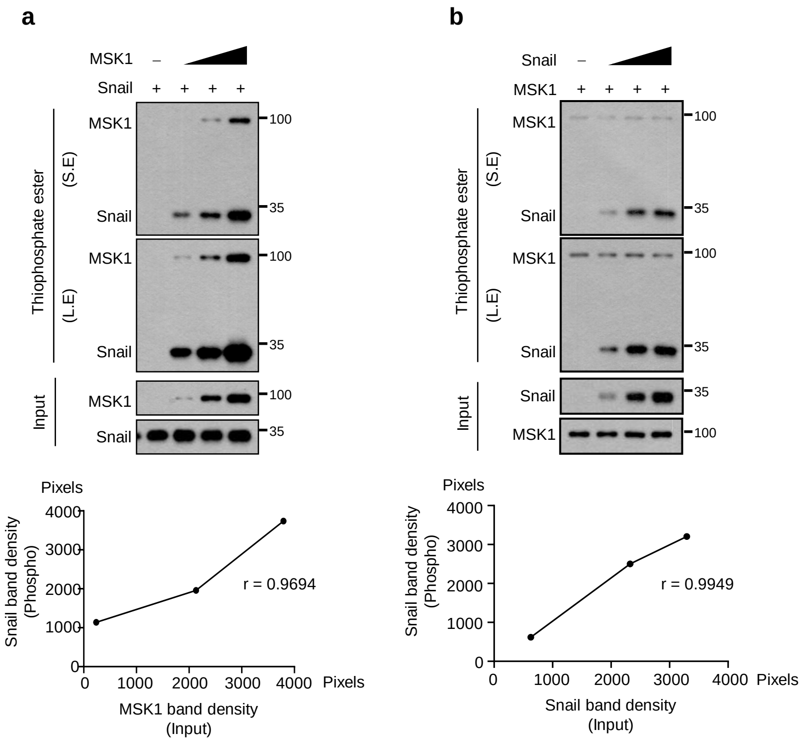

Supplementary Fig. 5. MSK1 phosphorylates Snail *in vitro*.

(a) *In vitro* kinase assays were performed by incubating increasing amounts of recombinant active MSK1 protein with recombinant Snail protein (a) or recombinant active MSK1 protein with increasing amounts of recombinant Snail protein (b) in the presence of ATP-γ-S. The resultant products were subjected to SDS-polyacrylamide gel electrophoresis and analyzed by immunoblotting using an anti-Thiophosphate ester antibody. S.E.: short exposure, L.E.: long exposure.

Supplementary Figure 6

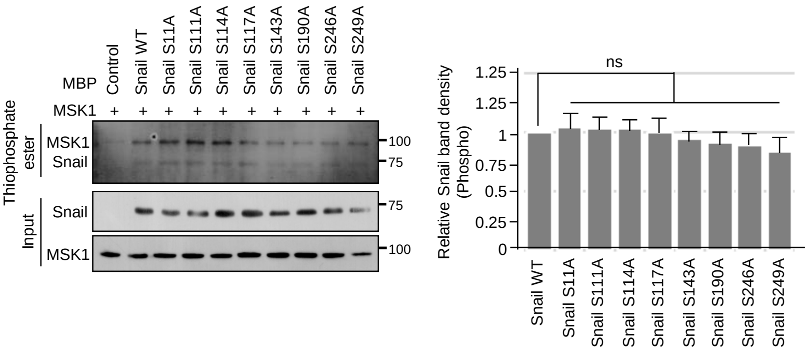

Supplementary Fig. 6. MSK1 phosphorylates Snail mutants *in vitro*.

In vitro kinase assays were performed by incubating recombinant active MSK1 protein with Purified MBP-fused WT-Snail or Snail serine mutants (S11A, S111A, S114A, S117A, S143A, S190A, S246A, or S249A) in the presence of ATP-γ-S. The resultant products were subjected to SDS-polyacrylamide gel electrophoresis and analyzed by immunoblotting using an anti-Thiophosphate ester antibody.

Supplementary Figure 7

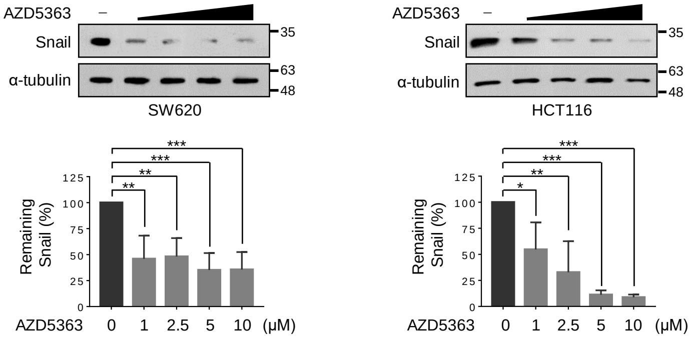

Supplementary Fig. 7. Effect of AZD5363 on Snail expression in SW620 and HCT116 cells. SW620 or HCT116 cells were treated with increasing amounts (1 to 10 μM) of AZD5363 for 24 h. Cell lysates were immunoblotted with anti-Snail antibody (upper). The data are representative of three independent experiments and relative Snail levels were quantified using ImageJ software (lower). *, $P < 0.05$: **, $P < 0.01$: ***, $P < 0.001$ as determined by *t*-test.

Supplementary Figure 8

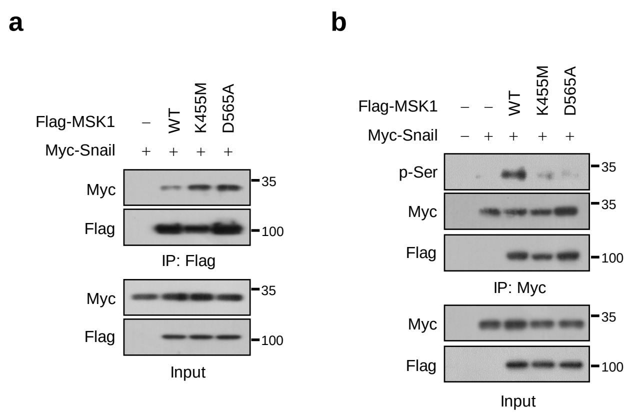

Supplementary Fig. 8. K455M-MSK1 and D565A-MSK1 can interact with Snail, but cannot phosphorylate it.

Flag-tagged WT-MSK1, K455M- or D565A-MSK1 was co-transfected with Myc-Snail into HEK293T cells, and then treated with 10 μM MG132 for 6 h. (a) Cell lysates were immunoprecipitated using an anti-Flag antibody and then analyzed by immunoblotting using an anti-Myc antibody. (b) Cell lysates were immunoprecipitated using an anti-Myc antibody and then analyzed by immunoblotting using an anti-phospho-Ser (p-Ser) antibody.

Supplementary Figure 9

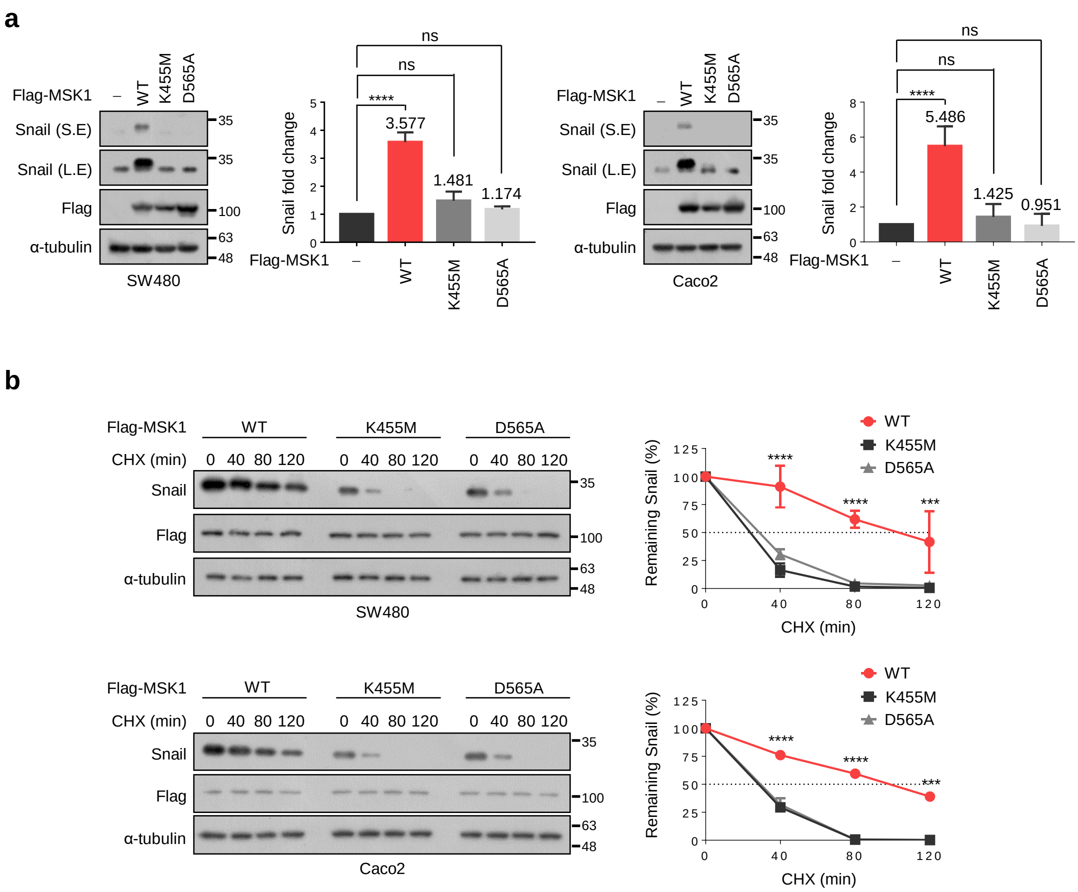

Supplementary Fig. 9. K455M-MSK1 and D565A-MSK1 cannot increase Snail protein stability in CRC cells.
(a) WT-MSK1, K455M- or D565A-MSK1 was transfected into SW480 or Caco2 cells. Cell lysates were immunoblotted with the indicated antibodies (left). The data are representative of three independent experiments and relative Snail levels were quantified using ImageJ software (right). ****, $P < 0.0001$ as determined by *t*-test. (b) WT-MSK1, K455M- or D565A-MSK1 was transfected into SW480 or Caco2 cells in the presence of CHX (100 µg/ml) for the indicated times. Cell lysates were immunoblotted by antibodies as indicated (left). The data were quantified using ImageJ software (right). For normalization, α-tubulin expression was used as a control. ***, $P < 0.001$: ****, $P < 0.0001$ as determined by *t*-test.

Supplementary Figure 10

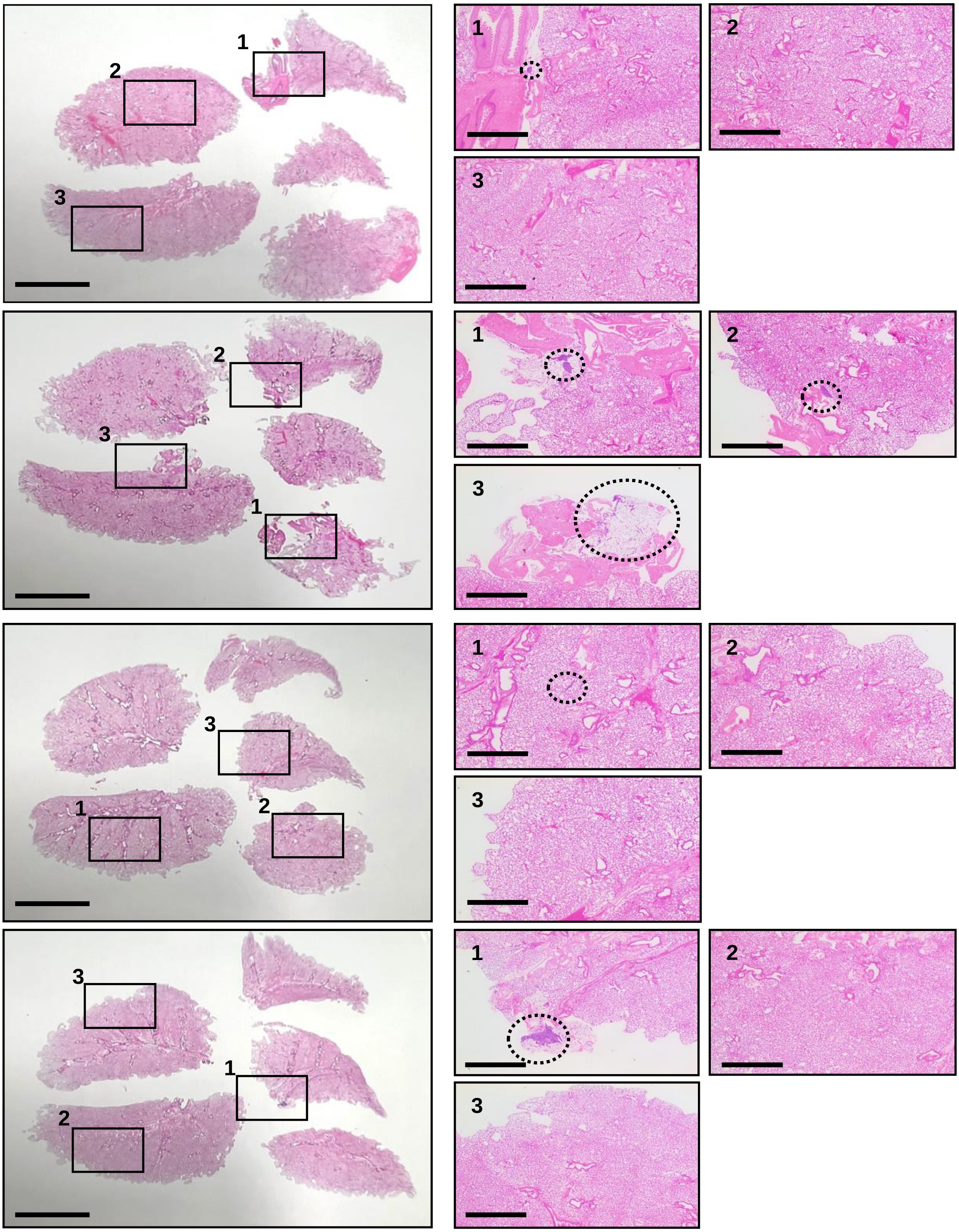

Supplementary Fig. 10. HE staining of whole lung sections after injection of control and WT-MSK1, K455M-MSK1 or D565A-MSK1 overexpressing SW480 cells.
Representative HE-stained images of the entire lung 45 days after tail-vein injection of control and WT-MSK1 or MSK1 mutants (K455M, D565A)-overexpressing SW480 cells in male BALB/c/nude mice. The images illustrate the histological features and differences between the conditions. Scale bar in the whole lung: 3 mm, Scale bar in the magnified image: 100 µm.

Supplementary Figure 11

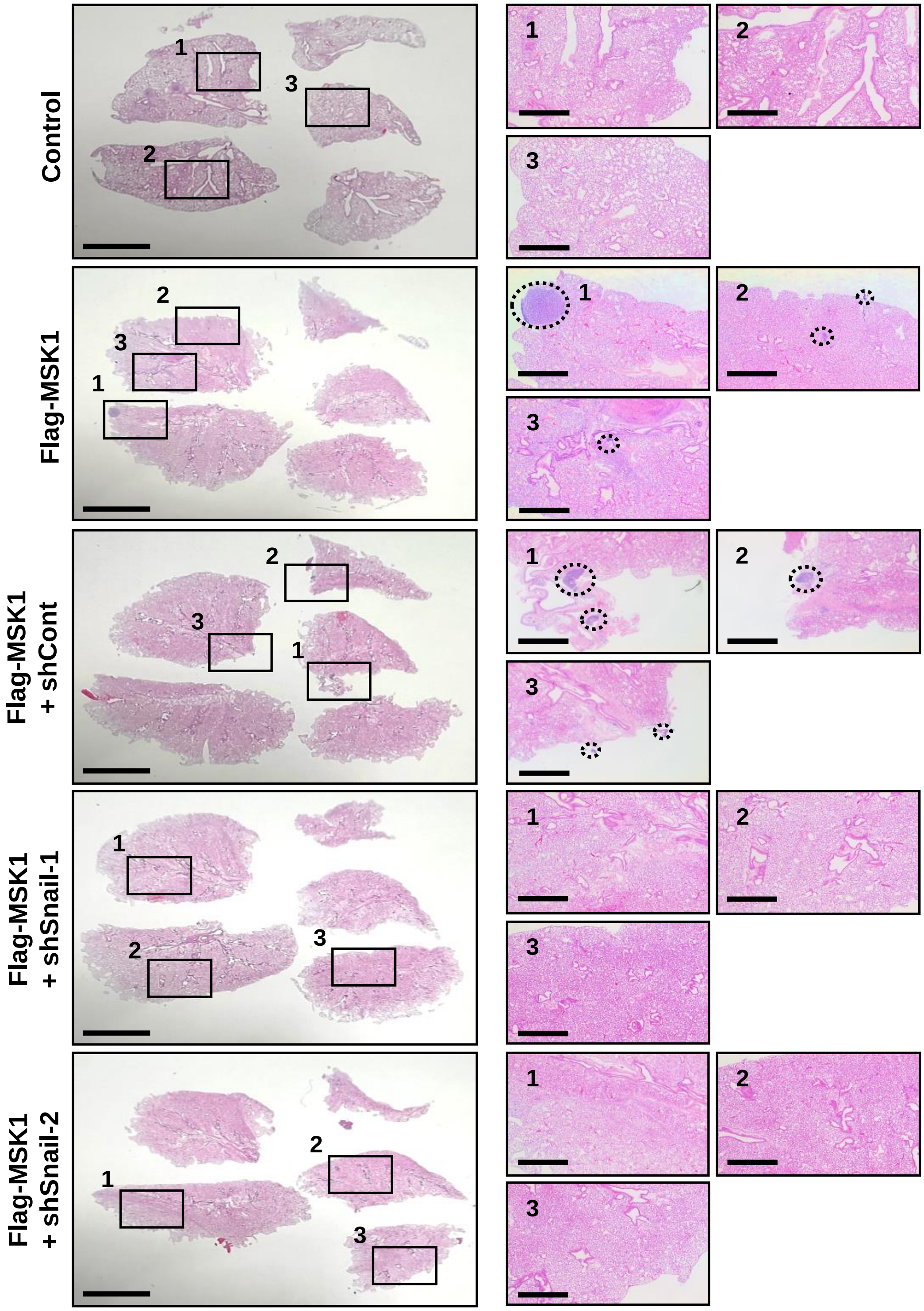

Supplementary Fig. 11. HE staining of whole lung sections after injection of Snail-depleted MSK1-overexpressing SW480 or control cells.

Representative HE-stained images of the entire lung 45 days after tail-vein injection of Snail-depleted MSK1-overexpressing SW480 or control cells. The images illustrate the histological features and differences between the conditions. Scale bar in the whole lung: 3 mm, Scale bar in the magnified image: 100 µm.

Supplementary Figure 12

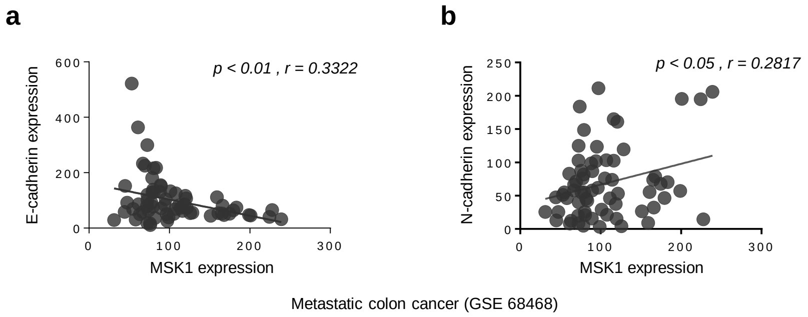

Supplementary Fig. 12. Correlation between MSK1 mRNA expression levels and mRNA levels of representative EMT marker genes (E-cadherin and N-cadherin) in CRC patients.

Co-expression of E-cadherin (a) or N-cadherin (b) with MSK1 mRNAs was analyzed colorectal cancer patients (GSE 68468). Two-tailed non-parametric Spearman correlation was used. The linear regression line is shown in black.

Supplementary Figure 13

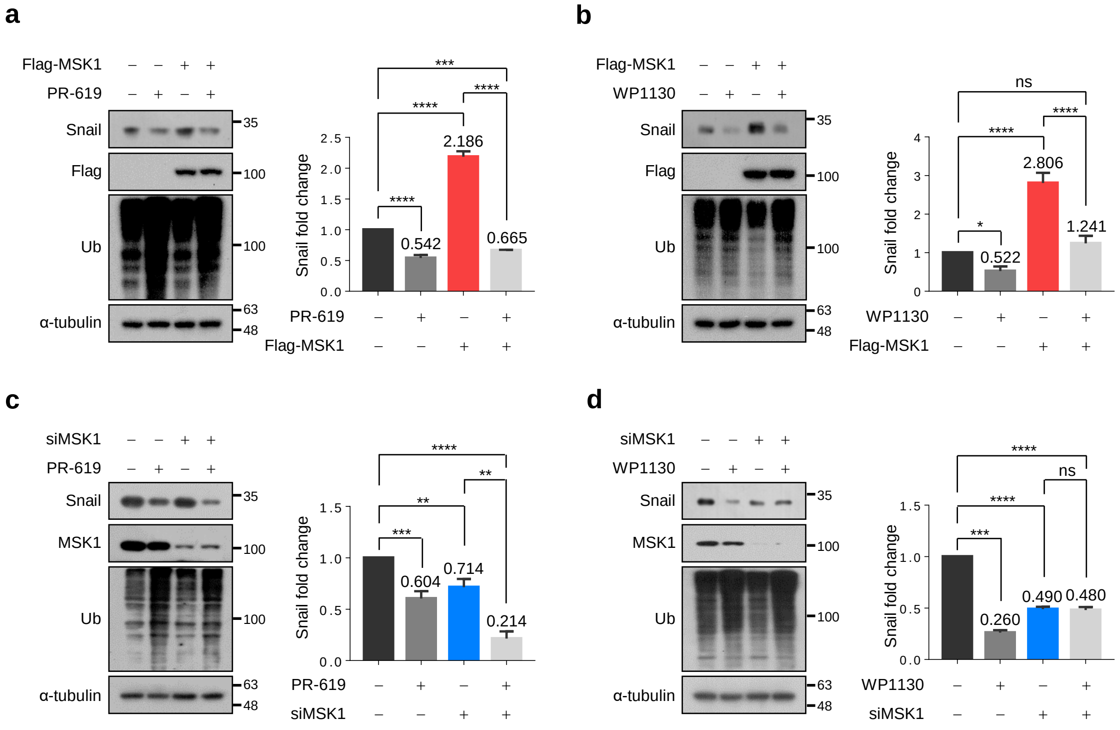

Supplementary Fig. 13. Screening of DUBs involved in MSK1-induced increase in Snail protein stability.

(a, b) MSK1 was transfected into HEK293T cells, and then the cells were treated with 10 μM PR-619 (a) or 3 μM WP1130 (b) for 3 h, respectively. Cell lysates were immunoblotted with the indicated antibodies (left). The data are representative of three independent experiments and relative Snail levels were quantified using ImageJ software (right). *, *P* < 0.05: ****, *P* < 0.0001 as determined by *t*-test. (c, d) MSK1-specific siRNA was transfected into HEK293T cells, and then the cells were treated with 10 μM PR-619 (c) or 3 μM WP1130 (d) for 3 h, respectively. Cell lysates were immunoblotted with the indicated antibodies (left). The data are representative of three independent experiments and relative Snail levels were quantified using ImageJ software (right). **, *P* < 0.01: ***, *P* < 0.001: ****, *P* < 0.0001 as determined by *t*-test.

Supplementary Figure 14

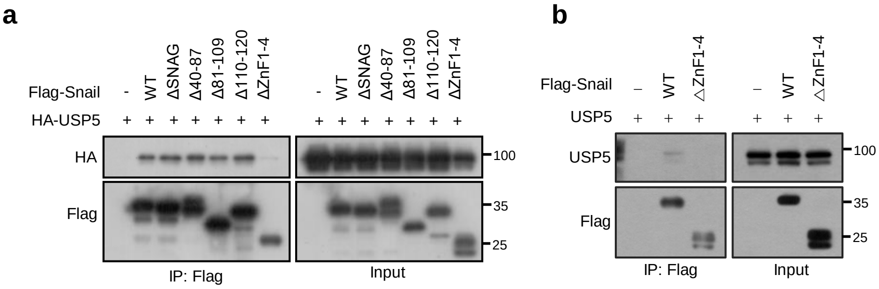

Supplementary Fig. 14. The zinc finger domain of Snail is essential for its interaction with USP5. (a) Interaction between exogenous USP5 and Snail deletion mutants. HEK293T cells transfected with HA-USP5 and Flag-tagged respective Snail deletion mutants were immunoprecipitation with anti-Flag antibody and analyzed by western blot using anti-HA antibody. (b) *In vitro* interaction of USP5 with WT-Snail or Snail-ΔZnF 1-4. Recombinant USP5 was mixed with lysates from HEK293T cells transfected with Flag-tagged WT-Snail or Snail-ΔZnF 1-4, and the mixture was incubated at 30°C for 30 min. Immunoprecipitation was then performed using an anti-Flag and analyzed by western blot using anti-USP5 antibody

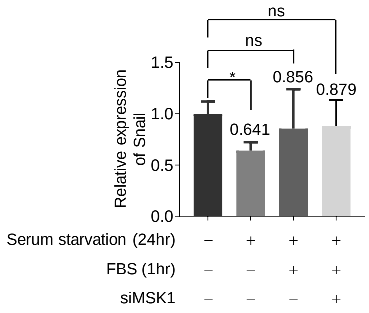

Supplementary Fig. 15. mRNA levels of Snail by serum stimulation after serum depletion in MSK1-depleted HCT116 cells .

MSK1-depleted HCT116 cells were incubated for 24 h in serum-free medium and then stimulated with DMEM media including 10% FBS for 1 h. mRNA was extracted from each cell lysate and mRNA level of Snail was analyzed by qRT-PCR. For normalization, GAPDH expression was used as a control.
